# Bacterioplankton Dynamics within a Large Anthropogenically Impacted Urban Estuary

**DOI:** 10.3389/fmicb.2015.01438

**Published:** 2016-01-26

**Authors:** Thomas C. Jeffries, Maria L. Schmitz Fontes, Daniel P. Harrison, Virginie Van-Dongen-Vogels, Bradley D. Eyre, Peter J. Ralph, Justin R. Seymour

**Affiliations:** ^1^Plant Functional Biology and Climate Change Cluster, University of Technology SydneySydney, NSW, Australia; ^2^Hawkesbury Institute for the Environment, Western Sydney UniversityPenrith, NSW, Australia; ^3^School of Geosciences, University of Sydney Institute of Marine Science, The University of SydneySydney, NSW, Australia; ^4^Sydney Institute of Marine ScienceMosman, NSW, Australia; ^5^Centre for Coastal Management, Southern Cross UniversityLismore, NSW, Australia

**Keywords:** microbial ecology, estuarine ecology, metagenomics, anthropogenic impacts, environmental pollutants, environmental microbiology, microbiome, eutrophication

## Abstract

The abundant and diverse microorganisms that inhabit aquatic systems are both determinants and indicators of aquatic health, providing essential ecosystem services such as nutrient cycling but also causing harmful blooms and disease in impacted habitats. Estuaries are among the most urbanized coastal ecosystems and as a consequence experience substantial environmental pressures, providing ideal systems to study the influence of anthropogenic inputs on microbial ecology. Here we use the highly urbanized Sydney Harbor, Australia, as a model system to investigate shifts in microbial community composition and function along natural and anthopogenic physicochemical gradients, driven by stormwater inflows, tidal flushing and the input of contaminants and both naturally and anthropogenically derived nutrients. Using a combination of amplicon sequencing of the 16S rRNA gene and shotgun metagenomics, we observed strong patterns in microbial biogeography across the estuary during two periods: one of high and another of low rainfall. These patterns were driven by shifts in nutrient concentration and dissolved oxygen leading to a partitioning of microbial community composition in different areas of the harbor with different nutrient regimes. Patterns in bacterial composition were related to shifts in the abundance of *Rhodobacteraceae, Flavobacteriaceae, Microbacteriaceae, Halomonadaceae, Acidomicrobiales*, and *Synechococcus*, coupled to an enrichment of total microbial metabolic pathways including phosphorus and nitrogen metabolism, sulfate reduction, virulence, and the degradation of hydrocarbons. Additionally, community beta-diversity was partitioned between the two sampling periods. This potentially reflected the influence of shifting allochtonous nutrient inputs on microbial communities and highlighted the temporally dynamic nature of the system. Combined, our results provide insights into the simultaneous influence of natural and anthropogenic drivers on the structure and function of microbial communities within a highly urbanized aquatic ecosystem.

## Introduction

Estuaries are among the most urbanized coastal ecosystems (Line and White, 2007) and as a consequence experience substantial environmental pressures, including habitat loss, decreased biodiversity, harmful algal blooms, anoxia, and contamination by sewage, pesticides, polycyclic aromatic hydrocarbons, heavy metals and other organic and inorganic pollutants (Birch et al., 1999; Diaz and Rosenberg, 2008). While these anthropogenically derived pressures have negative influences felt throughout entire estuarine food webs, the most prominent impacts often occur among populations of planktonic and sediment-bound microorganisms. Due to their sensitive and rapid responses to chemical perturbations, estuarine microbial communities often simultaneously act as sentinels of environmental impact and contributors to further deterioration of habitat health (Sun et al., 2012). While dramatic and conspicuous changes in estuarine biogeochemistry (e.g., anoxia, hydrogen sulfide evolution) often occur as a consequence of shifts in ecosystem microbiology (Cole, 1999; Crump et al., 2007; Breitburg et al., 2010; Abell et al., 2013, 2014), the microbial ecology underpinning these changes is often not well understood.

Estuaries typically host high microbial diversity and display substantial spatiotemporal heterogeneity in microbial abundance, activity and composition as a consequence of natural gradients of physical (e.g., light, salinity, and temperature) and chemical (e.g., inorganic and organic nutrients) conditions between the watershed and the mouth of the estuary (Crump et al., 2007; Fortunato et al., 2012). In addition to natural variation in environmental parameters, anthropogenic impacts such as increased nutrient input (eutrophication) and contamination by substances such as hydrocarbons and industrial effluent can lead to shifts in microbial communities in urbanized estuaries (Gillan et al., 2005; Vieira et al., 2008; Gregoracci et al., 2012; Sun et al., 2012, 2013) with implications for both ecosystem and human health. Microbial communities are extremely sensitive to rapid changes in the environment and can be used as indicators of stress (Paerl, 2006; Sun et al., 2012) as changes in the relative abundance of specific taxa or functional genes can be indicative of shifts in the physicochemical dynamics within estuaries and coastal systems (Smith et al., 2010; Fortunato et al., 2012, 2013; Gregoracci et al., 2012).

Within estuaries, natural freshwater inputs strongly influence the chemical, physical, and biological characteristics of the ecosystem, and salinity gradients often drive strong shifts in the structure of microbial communities, with discrete assemblages forming in zones of different salinity (Bouvier and Del Giorgio, 2002; Crump et al., 2004; Kirchman et al., 2005; Smith et al., 2010; Fortunato et al., 2012, 2013; Campbell and Kirchman, 2013). Due to the high human population densities that often occur near to estuaries, anthropogenic pressures also create significant spatial and temporal heterogeneity in physical and chemical conditions within estuaries. The dynamics of these anthropogenic inputs can be expressed either as spatial gradients along an estuary, such as in the case of concentrated inputs of agricultural or urban contamination at the top of the estuary, or as localized, and often pulse, inputs, leading to hotspots of contamination. Anthropogenic influences can include the input of organic and inorganic nutrient rich stormwater and sewage run-off (Beck and Birch, 2012), agricultural run-off, which can be rich in either inorganic nutrients from fertilizers or toxins from pesticides (Vieira et al., 2008; Gregoracci et al., 2012), industrial waste enriched in heavy metals and other toxins (McCready et al., 2006) or thermal pollution associated with the cooling water from powerplants and other large industry (Shiah et al., 2006). Microbial responses to these impacts can lead to altered and often unbalanced nutrient cycles, anoxic conditions, blooms of harmful algae (Paerl, 1997, 2006; Anderson et al., 2002) and increases in enteric and endemic pathogen concentrations (Hsieh et al., 2007). On the other hand, microbial populations may also assist in remediating estuarine systems, by rapidly degrading contaminants including hydrocarbons and fertilizers (Head et al., 2006). However, the inherent spatial and temporal variability in microbial community composition and function that occurs in estuaries means that these “positive” and “negative” ecosystem services provided by microbial assemblages will likely show substantial heterogeneity in space and time. Therefore, understanding the microbial ecology of urban estuaries is fundamentally important to monitoring the occurrence, distribution and fate of pollutants.

Here, we used a model-urbanized estuary, Sydney Harbor, to examine microbial dynamics over space and time within an anthropogenically impacted ecosystem. More than 85% of the harbor's catchment area is urbanized, with almost one quarter of the total population of Australia living in the surrounding area (Birch et al., 1999). Historically, the most urbanized areas are located in the western region of Sydney Harbor, where multiple negative impacts have been experienced, including elevated levels of pesticides, polycyclic aromatic hydrocarbons, nutrients, heavy metals, and suspended material as well as regular algal blooms and periods of anoxia (Birch et al., 1999; McCready et al., 2000, 2006; Birch and Rochford, 2010; Beck and Birch, 2012; Hedge et al., 2014).

Precipitation regimes strongly influence the hydrology and biogeochemistry of the Sydney Harbor estuary system, which remains well-mixed under low- or no-rainfall events, but experiences substantial horizontal stratification following high-rainfall events (Birch et al., 1999), primarily as a consequence of point-source inputs from localized stormwater runoff points (Beck and Birch, 2012). As a consequence, a qualitative correlation between rainfall events and a range of ephemeral environmental episodes, including algal blooms and periods of anoxia, have been recorded in the estuary (Birch et al., 1999; Beck and Birch, 2012). It is well established that rainfall results in the allochtonous input of both naturally derived nutrients from the catchment area, and nutrients from anthropogenic sources such as fertilizer and sewage (Hedge et al., 2014). Indeed sewage has been responsible for over 50% of the total nitrogen and phosphorous in this system (Birch et al., 2010) and urban run-off and sewage overflows are two major contributor's to nutrient input into the system (Hedge et al., 2014). However, despite the ecological, economic and intrinsic national importance of the Sydney Harbor estuary and an increasing interest in the ecology of this habitat (Hedge et al., 2014) the microbial ecology of this system remains virtually unexplored and we lack any perception of how the diversity and ecological function of Sydney Harbor's microbiota responds to ongoing anthropogenic pressures. Here we address this knowledge gap by applying amplicon sequencing and shotgun metagenomic approaches to investigate the microbial ecology of a model urbanized estuary within the context of spatiotemporal heterogeneity in environmental variables. Therefore, we hypothesize that structure of the microbial community inhabiting the Sydney Harbor estuary will be highly variable in space and time as a consequence of both natural and anthropogenically-driven environmental heterogeneity.

## Materials and methods

### Study area and water collection

**Figure 1:**
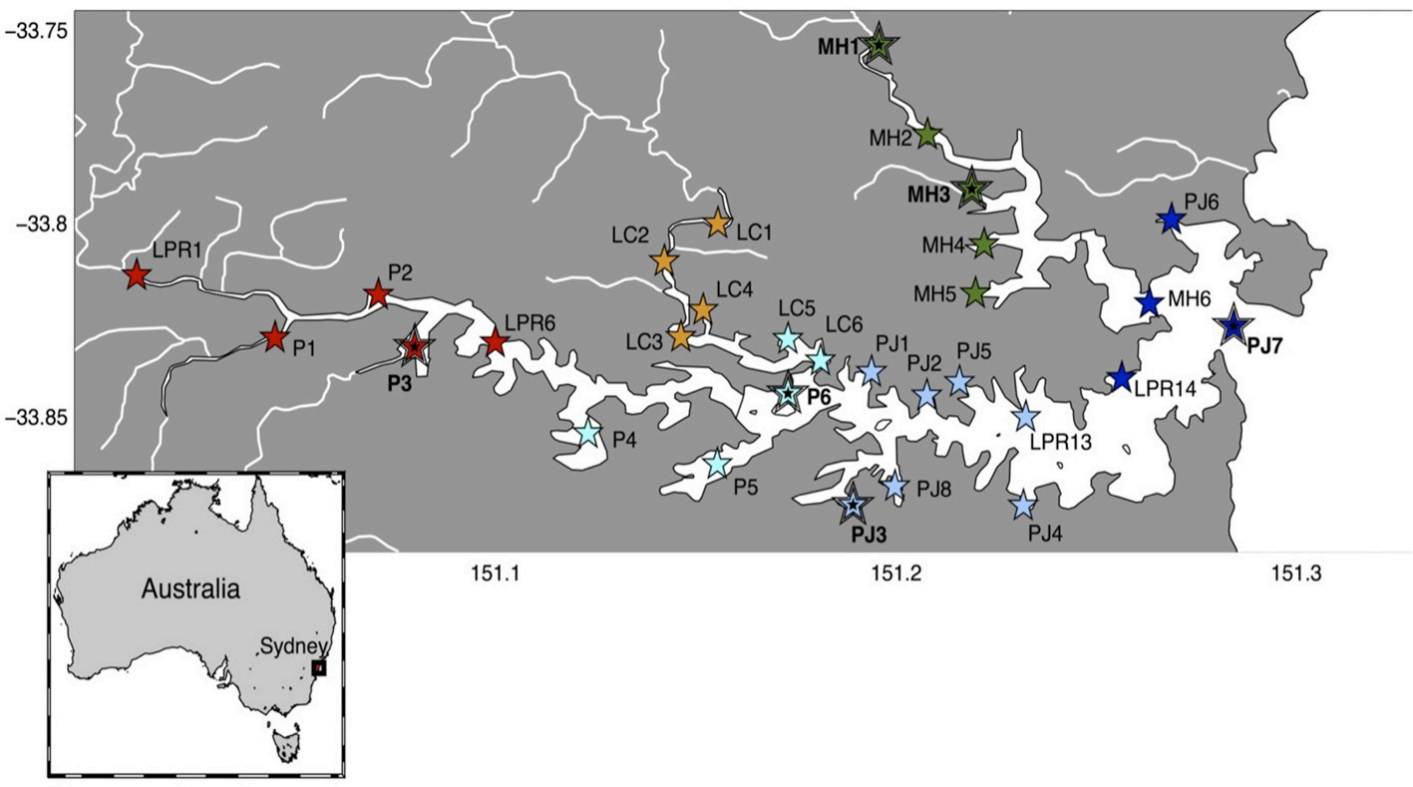
**Sampling locations within Sydney Harbor**. Color of symbols indicates geographic regions of the estuary with large filled symbols indicating samples for which shotgun metagenomes were analzyed in addition to amplicon 16S rDNA libraries. Parramatta River, red symbols; Lane Cove, orange symbols; Middle Harbor, dark green symbols; Western-Harbor, cyan symbols; Eastern-Harbor, pale blue symbols; and Marine/Harbor Heads, dark blue symbols.

The Sydney Harbor estuary has a total area of 480 km^2^, and it is among the most highly urbanized regions of the Australian coast (Birch et al., 2010). The Harbor's average depth is approximately 10 m (maximum 46 m), and maximum tidal range is 2.1 m (Hatje et al., 2003), with flushing times that vary from < 1 day in the mouth up to 225 days in the uppermost regions of the estuary (Das et al., 2000). Land-use surrounding the Sydney Harbor estuary also varies, with more commercial and industrial areas located in the inland, western parts of the estuary (Birch et al., 2010), relative to the more pristine and marine conditions near to the estuary mouth in the east (Figure 1).

**Figure 2:**
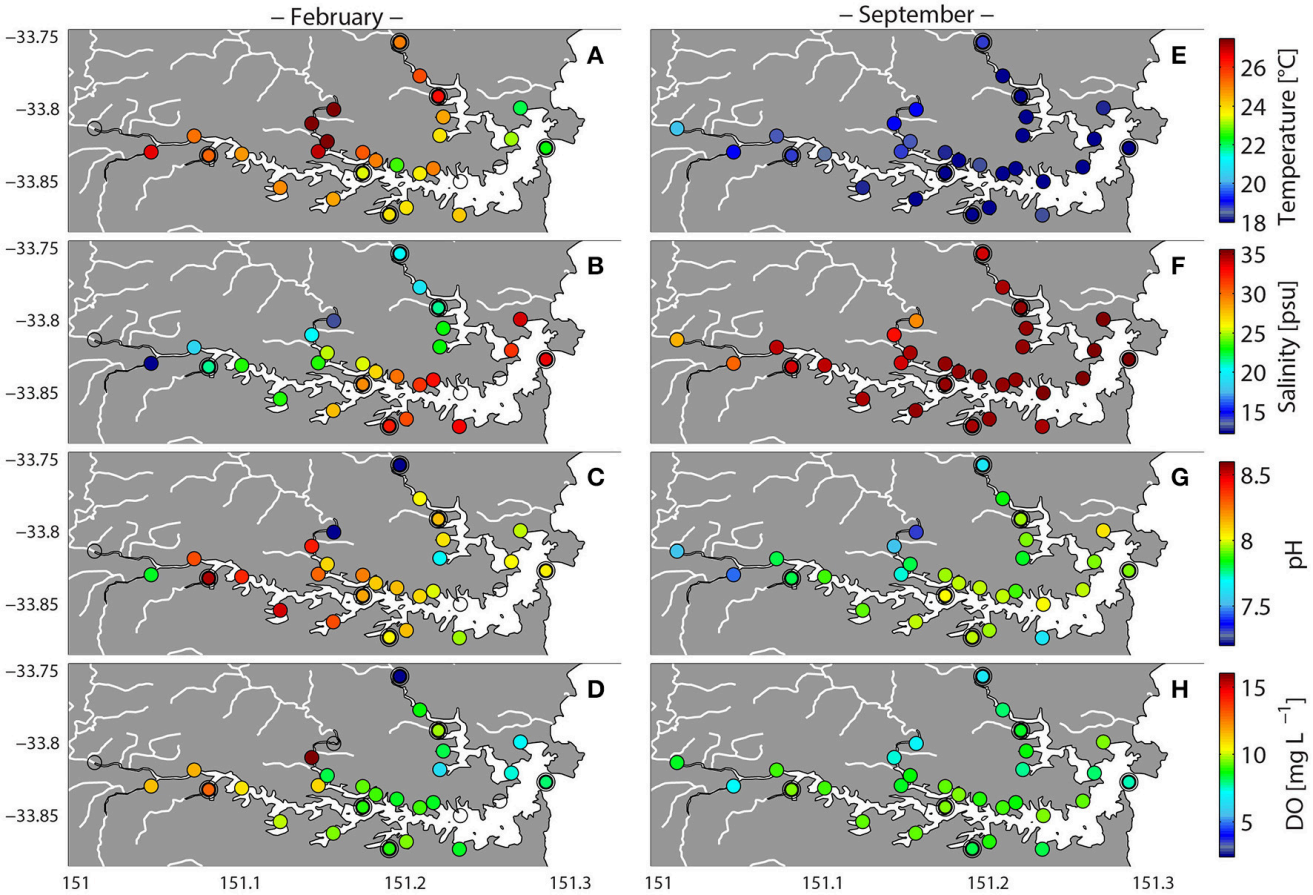
**Variability in environmental variables in Sydney Harbor during high-rainfall (February 2013) and no rainfall (September 2013)**. Double circled symbols represent sites for which shotgun metagenomes were analyzed. Temperature **(A,E)**, Salinity **(B,F)**, pH **(C,G)**, dissolved oxygen (DO) **(D,H)**.

We examined patterns in the composition and function of microbial assemblages across 30 sites spanning the 30 km length of the estuary (Figure 1) from Parramatta weir in the western brackish region of the estuary, where salinity levels are riverine (e.g., 12 ppt during rainfall), to the mouth of the estuary (Sydney Heads), where marine conditions are experienced (e.g., 34 ppt; Figure 2). These sites covered six main regions of the estuary, and were defined geographically as the Parramatta River (*n* = 5), Lane Cove (*n* = 4), Middle Harbor (*n* = 5), Western-Harbor (*n* = 5), Eastern-Harbor (*n* = 7) and Marine/Harbor Heads (*n* = 4) regions (Figure 1). Sample collection was timed to capture the dynamics of bacterial communities during two contrasting precipitation periods: a moderately high rainfall period in February (average monthly total 165.4 mm; 184 mm in the 2 weeks preceding sampling) and a low rainfall period in September (average monthly total 32.2 mm, 0 mm in the 2 weeks preceding sampling; Australian Bureau of Meteorology). We recognize that these sampling periods provide snap-shots of different precipitation regimes in this environment and are not suitable for predicting the long-term temporal heterogeneity of the estuary. Given the disparate environmental conditions of these time-points (discussed below), they were representative examples of contrasting ecosystem states in the estuary.

### Water quality and nutrient analysis

Physical and biological properties were measured at each site using a multi-parameter water quality probe (YSI-6600, Yellowstone Instruments, USA) fitted with; conductivity, temperature, depth, pH, O_2_, turbidity, & chlorophyll sensors. For nutrient analysis, 5 ml unfiltered sample was immediately collected for Total Nitrogen (TN) and Total Phosphorus (TP) and placed into a 10 ml polycarbonate vial, 80 ml was filtered immediately through a polycarbonate 0.45 μm cellulose acetate syringe filter (Whatman) for dissolved nutrient analysis including mono-nitrogen oxides, nitrite, nitrate, phosphates, ammonium, silicate (NO_x_, ${\text{NO}}_{2}^{-}$, ${\text{NO}}_{3}^{-},$ ${\text{PO}}_{4}^{3-},$ ${\text{NH}}_{4}^{+}$, and Si respectively), Total Dissolved Nitrogen (TDN) and Total Dissolved Phosphorous (TDP). All vials were placed on ice in the dark and frozen (−30°C) prior to analysis (within 3 months).

Nutrients were analyzed using flow injection analysis on a LaChat 8500 instrument. Total Nitrogen and Total Phosphorus (TN/TP) and TDN/TDP were prepared jointly using the modified alkaline peroxidisulfate autoclave digestion described by Maher et al. (2002). NO_x_, ${\text{NH}}_{4}^{+}$, ${\text{PO}}_{4}^{3-}$, and Si were analyzed using standard methods 4500-N0${}_{3}^{-}$ G., 4500-NH_3_ H., 4500-P, and 4500-S_i_O_2_, F respectively (APHA, 2005). ${\text{NO}}_{2}^{-}$ was analyzed using the NO_x_ method without cadmium reduction. Total suspended solids (TSS) were measured gravimetrically after drying at 105°C using a 25 mm GF/F filter (Whatman), Standard Method 2540 D, (APHA, 2005). Further details of nutrient analysis can be found in Eyre (2000).

### DNA extraction and 16S rRNA analysis

Two liter near-surface water samples (~0.5 m depth) water were filtered onto 0.2 μm polycarbonate membrane filters (Millipore), and filters were stored at -20°C until DNA extraction. DNA was extracted using a bead beating and chemical lysis kit (MOBIO PowerWater, Carlsbad, CA, USA), according to the manufacturer's instructions. Genomic DNA concentrations were measured using a Qubit 2.0 fluorometer (Invitrogen, Carlsbad, CA, USA).

16S rRNA amplicon pyrosequencing was used to profile the composition of bacterial communities. Briefly, DNA samples were amplified with the 16S rRNA universal Eubacterial primers 803F (5′-attagataccctggtagtc-3′) and 1392R (5′-acgggcggtgtgtRc-3′; Engelbrektson et al., 2010) using the following cycling conditions: 95°C for 3 min; 25 cycles of 95°C for 30 s, 55°C for 45 s and 72°C for 90 s; followed by a final extension at 72°C for 10 min. Amplicons were subsequently sequenced on the 454 platform using titanium chemistry (Roche) at the Australian Centre for Ecogenomics (Queensland, Australia). DNA sequences were processed using the Quantitative Insights Into Microbial Ecology (QIIME) pipeline (Caporaso et al., 2010b) as previously described for 454 data (Gibbons et al., 2013). Briefly, DNA sequences were de-multiplexed and reads shorter than 200bp, with a quality score < 25, or containing homopolymers exceeding 6bp were discarded. The 16S rDNA data was rarefied to an equal number of sequences per sample (1563) and thus normalized for differences in sequencing depth. Operational Taxonomic Units were defined at 97% sequence identity using UCLUST (Edgar, 2010) and assigned taxonomy against the Greengenes database (version 13_5) (McDonald et al., 2012) using BLAST (Altschul et al., 1990). Chimeric sequences were detected using ChimeraSlayer (Haas et al., 2011) and filtered from the dataset. For beta-diversity analyses, representative sequences were aligned using PyNAST (DeSantis et al., 2006; Caporaso et al., 2010a) and the resultant phylogenetic tree, constructed using FastTree (Price et al., 2010), was used to calculate the weighted UniFrac distance between samples (Lozupone and Knight, 2005).

### Shotgun metagenomes

Complete environmental DNA (metagenome) analyses were carried out on six samples, which were chosen as representative samples from each of the six Sydney Harbor regions described above (MH1, MH3, P3, P6, PJ3, and PJ7) during the February sampling (Figure 1), when environmental variables and community composition (as determined by 16S rRNA amplicon sequencing) was observed to be most variable. DNA was sequenced using the Illumina HiSeq 2000 platform (2 × 100 bp; paired-ends, Australia Genome Research Facility Ltd (AGRF) in Victoria, Australia). Sequences were subsequently analyzed using the Meta Genome Rapid Annotation using Subsystems Technology (MG-RAST, version 3.5; Meyer et al., 2008; Glass et al., 2010). Quality control was performed using DRISEE (Duplicate Read Inferred Sequencing Error Estimation) (Keegan et al., 2012) to check for Artificial Duplicate Reads (ADRs), and estimating sequence error (Gomez-Alvarez et al., 2009) within the MG-RAST pipeline using default paramaters. After quality control a total of 1.5–2.3 Gbp were generated per metagenome. Clusters of proteins were based on a 60% identity level, and protein annotation was conducted using BLAT (Kent, 2002) and OpenMP (Wilke et al., 2014). Metabolic assignments were annotated using the SEED subsystems database (Overbeek et al., 2005). Matches with an *E*-value of 1 × 10^−5^ were considered significant using a minimum alignment of 30 bp of paired-end reads. All data were normalized to sequencing effort. The metagenomes can be accessed through MG-RAST under sample numbers 4550933.3 (site PJ3), 4550934.3 (site MH1), 4551435.3 (site MH3), 4551436.3 (site P3), 4551437.3 (site P6), and 4551749.3 (site PJ7).

### Statistical analyses

Statistical analyses were carried out in PRIMER + PERMANOVA software v.6 (Clarke, 1993; Clarke and Gorley, 2006; Anderson et al., 2008). Homogeneity of variance in our data was tested using PERMDISP (Anderson et al., 2008), and where homogeneity of variances was indicated, the chance of a Type I error was reduced by rejecting the null hypothesis at a probability of 0.01. Environmental data was log+1 transformed prior to analysis if homogeneity of variances was determined using draftsman plots, then standardized by subtracting the mean from each value and dividing by the standard deviation, and finally Euclidian distance was used to form similarity matrices. Biological data was square-root (SQRT) transformed prior to calculating the resemblance matrix using Bray-Curtis similarity. Environmental and biological data were then graphically represented using non-metric multi-dimensional scaling (MDS). A similarity percentage (SIMPER) was used to identify the phylogenetic groups and functional levels contributing mostly to the dissimilarity in each area of the harbor.

We also determined whether patterns seen in bacterial community and environmental data ordinations were similar using the RELATE analysis through a rank correlation value (Rho) and significance levels. Permutational multivariate analysis of variance (PERMANOVA) was used to determine significant dissimilarity within bacterial communities and environmental data, comparing February and September and six regions in the harbor. In order to show the environmental variables that best explained community patterns, we used BEST analysis, and a distance based linear modeling (DistLM)—using a stepwise procedure for adjusted *R*^2^—that selected the variables that most likely explained patterns in the biological data. This analysis was graphically represented by a distance-based redundancy analysis (dbRDA) plot. MDS, analysis of similarity (ANOSIM), DistLM, dbRDA analyses were also shown for each month separately.

To define the statistical relationships between all environmental variables and taxonomic groups identified in the 16S rRNA amplicon analysis, we used the Maximal Information-based Nonparamteric Exploration (MINE) algorithm (Reshef et al., 2011). MINE calculates the strength of the relationship between each individual variable (MIC score) in addition to descriptors of the relationship such as linearity and regression. Only variables with values for >50% of samples were included and the dataset was filtered to include only significant (*p* < 0.05) correlations. Results were visualized with Cytoscape V3 (Shannon et al., 2003).

For metagenomic analysis, functional reconstructions generated using MG-RAST were imported into the Statistical Analysis of Metagenomic Profiles (STAMP 2.0.8) package (Parks et al., 2014) to determine statistically significant differences among metagenomes. We conducted a Fisher's exact test (Rivals et al., 2007) with Benjamini FDR multiple correction to identify the significant different functional categories between two samples (Benjamini and Hochberg, 1995). The corrected *p*-values (*q*-values) were used and only *q*-values < 0.05 were reported (Parks and Beiko, 2010). Differences between proportions of two samples were shown within the 95% confidence intervals as positive and negative values for the most different functions using the Newcombe-Wilson method (Parks and Beiko, 2010). It must be noted that the metagenomic data was not replicated within each habitat, constraining the conclusions regarding site-driven differences in function. However, we feel that these samples provide valuable “snap-shots” of discrete communities within a highly heterogeneous system. Fisher's exact test uses a hypergeometric distribution of sequences drawn without replacement from a pair of metagenomic samples to generate a statistical significance value (Parks and Beiko, 2010) and is routinely applied for the pairwise comparison of metagenomes (e.g., Parks and Beiko, 2010; Mendes et al., 2014; Chen et al., 2015; Tout et al., 2015). Our results however should be interpreted as differences between discrete metagenomes/samples rather than ecologically distinct environments within the harbor.

## Results

### Environmental variables

Environmental parameters in Sydney Harbor were highly heterogeneous in February after a strong rainfall event, as reflected by patterns in salinity and water temperature (Figures 2A–D). Salinity levels varied between 34 at the mouth of the estuary to less than 13 at several up-river sites. Alternatively, salinity levels during September were much more homogenous, exceeding 28 at all sites (Figure 2F). During the February sampling, temperatures exceeding 26°C were observed in the western and upper river regions (Middle Harbor, Lane Cove, and Parramatta River), while in the eastern—central and marine regions of the estuary temperatures were between 22 and 24°C (Figure 2A). In September, temperatures were substantially more homogenous and near to 20°C throughout the entire estuary (Figure 2E).

**Figure 3:**
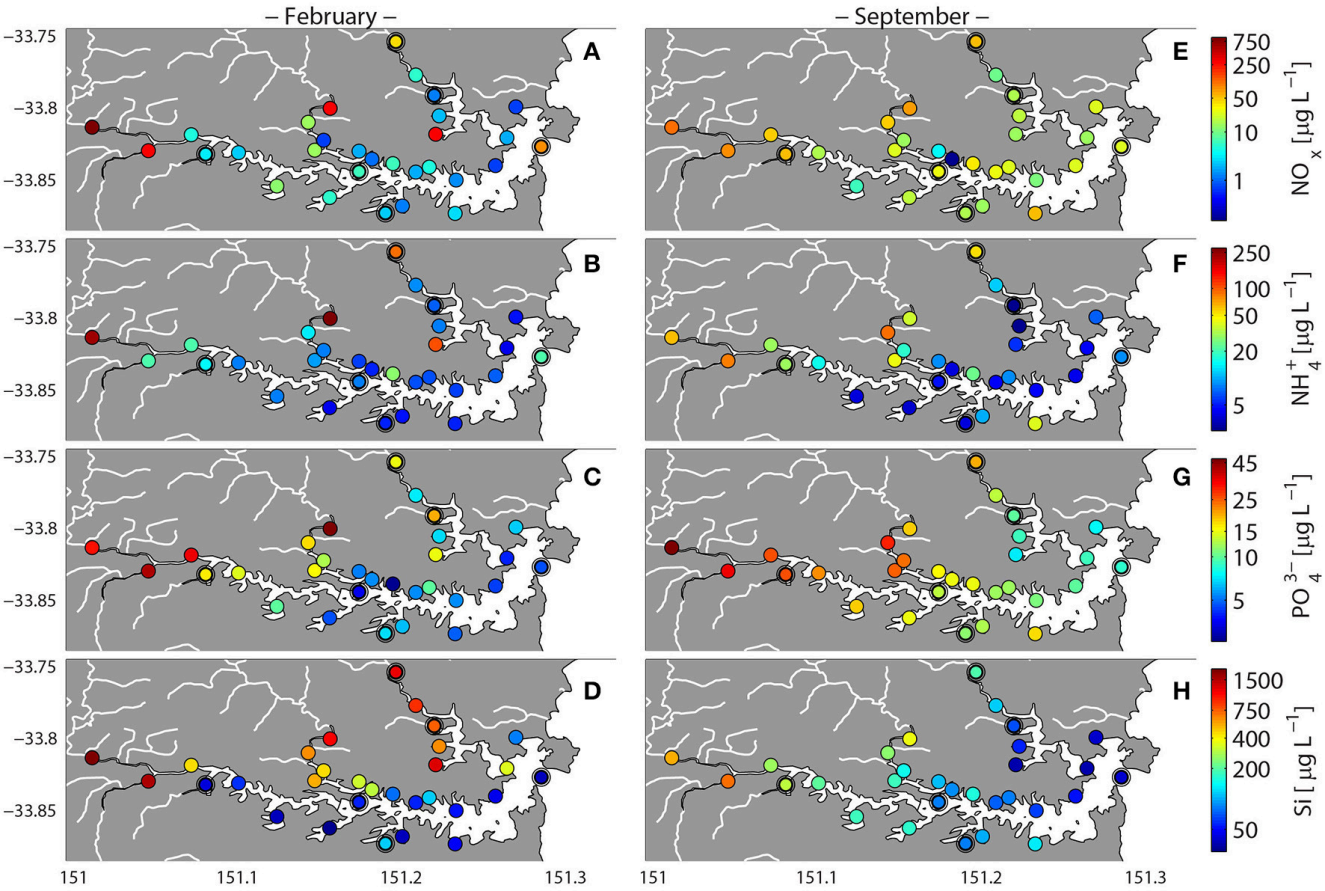
**Variability in nutrient concentrations in Sydney Harbor during high-rainfall (February 2013) and no rainfall (September 2013)**. Double circled symbols represent sites for which shotgun metagenomes were analyzed. Nitrate+nitrite **(A,E)**, Ammonium **(B,F)**, Phosphate **(C,G)**, Silicate **(D,H)**.

Dissolved oxygen (DO) and pH levels were generally higher across the estuary during February, but were more heterogeneous at this time, with some localized sites where DO levels were below 3 mg L^−1^ (e.g., site MH1; Figures 2C,D). In September, DO levels remained relatively consistent across the estuary, ranging between 6.6 and 9.5 mg L^−1^ (Figures 2G,H). Nutrient concentrations (NOx, ${\text{NH}}_{4}^{+}$, ${\text{PO}}_{4}^{3-}$, and Si) were also more heterogeneous across the estuary in February relative to September. During February, higher nutrient concentrations were generally observed in the western and upper river sites (Parramatta River, Lane Cove, and Middle Harbor; Figure 3). Within the Parramata River, Lane Cove River, and Middle Harbor regions of the estuary, the most inland (up-river) sites displayed higher levels of NOx, ${\text{NH}}_{4}^{+}$, ${\text{PO}}_{4}^{3-}$, and Si (Figure 3). While less variable than February, localized nutrient hotspots also occurred in September, often within the same locations as were observed in February. This was particularly true for phosphate, which was consistently elevated in the west and upper river sites. Notably phosphate levels were also elevated in the eastern-central harbor and marine-harbor heads regions in September (Figures 3C,G).

**Figure 4:**
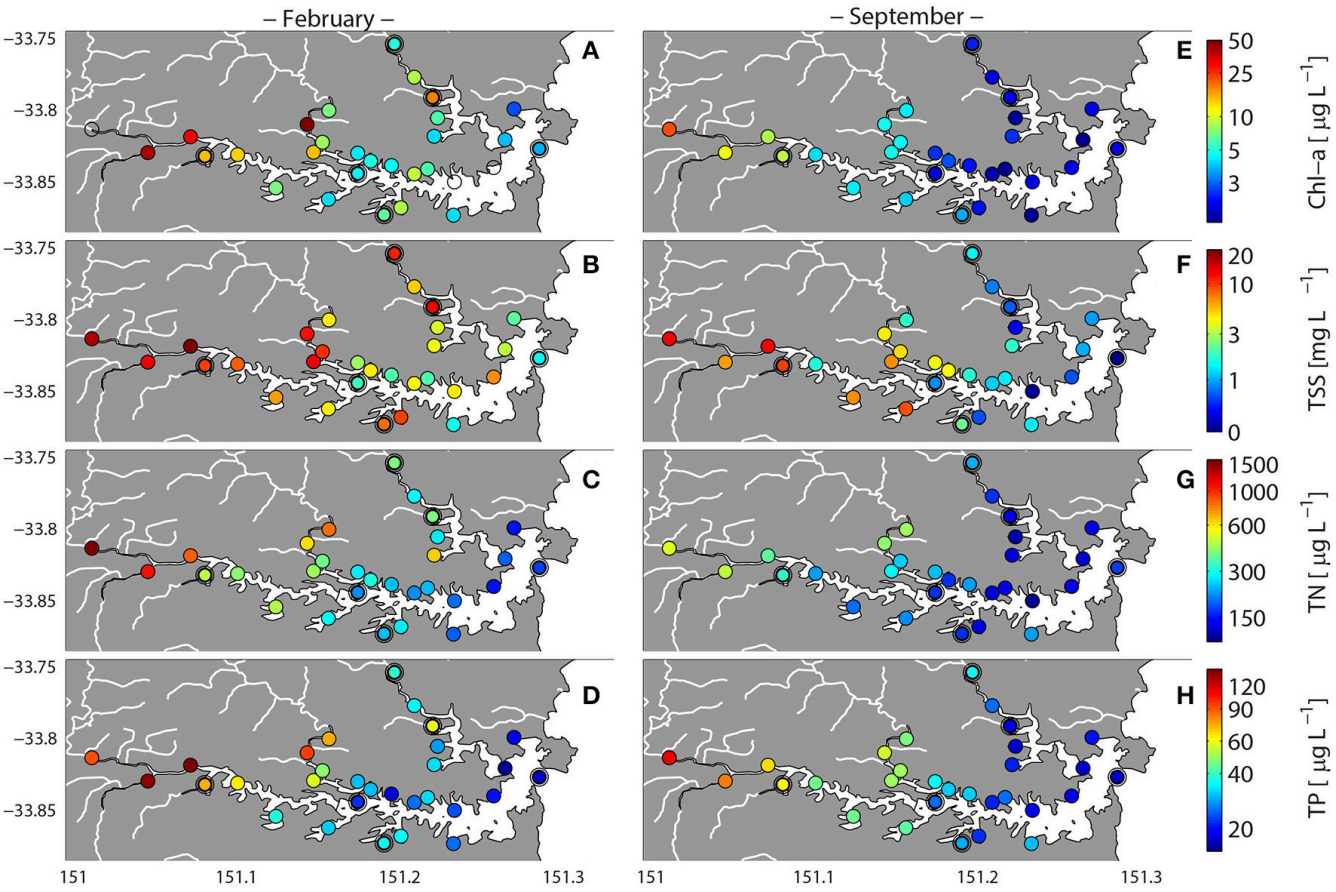
**Variation in total nutrient, chlorophyll, and suspended solid concentrations in Sydney Harbor during high-rainfall (February 2013) and no rainfall (September 2013)**. Double circled symbols represent sites for which shotgun metagenomes were analyzed. Chlorophyll-a **(A,E)**, Total Suspended Solids **(B,F)**, Total Nitrogen **(C,G)**, Total Phosphorus (TP) **(D,H)**.

**Figure 5:**
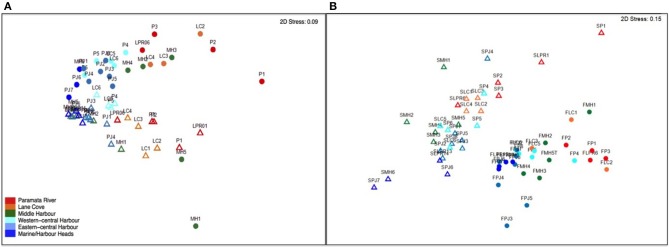
**MDS Ordination of (A) environmental variables and (B) microbial phylogenetic diversity and abundance in Sydney Harbor**. Colors designate geographic regions of the estuary. Circles, February 2013 (High Rainfall); Triangles, September 2013 (Low Rainfall).

Chlorophyll a (Chl a), Total Suspended Solids (TSS), TN, and TP all displayed the same spatial patterns, with hotspots observed in the west and upper river sites (Parramatta River, Western-central Harbor, Lane Cove, and Middle Harbor) during February, while during September, concentrations of suspended solids were elevated in the western region of the estuary relative to the east (Figure 4). When all environmental parameters were combined, the resultant ordination demonstrated a clear partitioning of samples between February and September. An exception to this was that during February the isolated and up-river sites at MH1 and MH5 displayed separation from all other sites (Figure 5A), suggesting that conditions within these two sites were physicochemically distinct from the rest of the Sydney Harbor ecosystem. These sites displayed reduced DO levels and increased concentrations of NOx and ${\text{NH}}_{4}^{+}$ and ${\text{PO}}_{4}^{3-}$ (Figures 2D, 3).

### Patterns in bacterial taxonomy

As observed in the ordination analysis for environmental variables, the bacterial community composition also displayed substantial spatiotemporal heterogeneity across the Sydney harbor estuary, with two clear clusters of samples corresponding to the February and September samples (R of RELATE Spearman = 0.779; Figure 5B). Spatial patterns corresponding with the different spatial regions of the estuary were also apparent within each cluster, with samples from the marine and eastern and middle harbor regions generally grouping together and those from the western, lane cove and riverine regions showing a high-degree of similarity (Figure 5B). Multidimensional scaling conducted for each month individually highlighted the partitioning of samples by region with ANOSIM analysis demonstrating that this grouping was more significant than the null distribution representing a random structure to community composition (*p* < 0.05) (Supplementary Material Figures 8, 9)

BEST analysis revealed that temperature, salinity, pH, dissolved oxygen and phosphate were the strongest drivers of differences between bacterial communities (Supplementary Material Table 1). DistML revealed that temperature was the main driver of community shifts over time, explaining 21% of variability. The combined influence of temperature, phosphate, pH, salinity, DO, and silicate explained 47% of the variability (Supplementary Material Table 1). Redundancy analysis revealed that temporal shifts in the bacterial community were driven by temperature and salinity (Supplementary Material Figure 1); and that the key environmental drivers for spatial shifts in the microbial community were silicate, phosphate, DO and pH (Supplementary Material Figure 1), where bacterial communities from western and upper river sites—MH1, MH5, LPR1, LC1, and P1—were positively related to silicate, and phosphate, and negatively to DO. In February, DistLM and RDA plots showed that salinity and pH were the main drivers of community shifts, whereas TDP and NOx were more important in September (Supplementary Material Figure 7).

**Figure 6:**
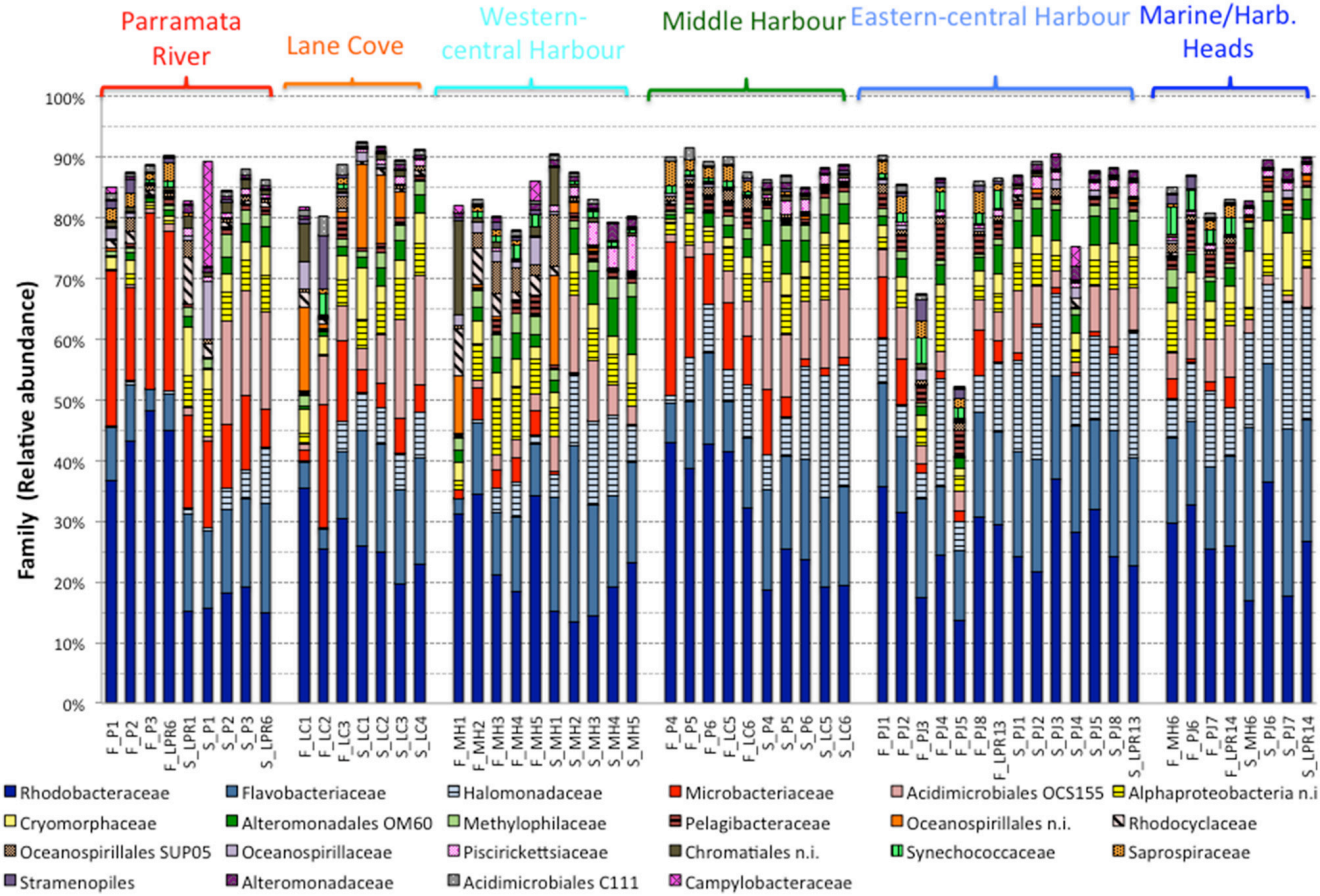
**Relative abundance of microbial taxa (family level) in Sydney Harbor**. Only families representing >0.1% abundance in any sample are shown. *F*, February 2013; S, September 2013.

*Rhodobacteraceae* were the most abundant bacterial family, followed by the *Flavobacteriaceae* and *Halomonadaceae*. However, the relative abundance of these groups shifted in both space and time (Figure 6). Common marine bacterial groups such as SAR11 and *Synechococcus* were also found in the Sydney Harbor estuary, not surprisingly occurring in highest abundance at the mouth of the harbor (MH6, PJ6, PJ7, and LPR14) relative to the western estuarine sites (LPR1, P1, LC1, and LC2), while groups such as the *Microbacteriaceae* were more relatively abundant in the western and upper-river sites, particularly during February (Figure 6).

### Temporal variability in community composition

SIMPER analysis revealed that Actinobacteria, Cyanobacteria and Bacteroidetes were the phyla that contributed the most to differences in bacterial community composition between the February and September sampling periods, and were together responsible for 39% of the temporal dissimilarity. On the other hand, Proteobacteria, the dominant phylum within the entire estuary, contributed to only 7.5% of total dissimilarity (Supplementary Material Table 2). Overall however, the relative abundance of phyla were less variable than at finer levels of taxonomic resolution, with individual families contributing to the dissimilarity between periods and showing larger shifts in relative abundance. For example, *Microbacteriaceae, Halomonadaceae, Rhodobacteraceae, Acidimicrobiales* OCS155, and *Flavobacteriaceae* contributed the most to differences in bacterial community composition between February and September, explaining 14.2% of dissimilarity (Supplementary Material Table 3).

Bacterial communities in the Parramatta River, upper-river and western-central harbor sites (i.e., the sites furthest from the marine conditions at the mouth of the estuary) displayed the strongest temporal shifts. The community shifted from one dominated by *Rhodobacteraceae* and *Microbacteriaceae* in February, to a community dominated by *Halomonadaceae, Acidimicrobiales* OCS155, and *Flavobacteriaceae* during September. In these three regions, there was also a notable shift in the ratio of *Rhodobacteraceae* to *Flavobacteriaceae* between February and September, with a higher relative proportion of *Flavobacteriaceae* observed during the dry period in September. Notably, these substantial shifts in bacterial community composition differed from those observed in the eastern sites of the harbor, where the bacterial assemblage was less variable between the two sampling periods (Figure 6).

### Spatial variability in community composition

Within individual sampling periods, there were clear biogeographic shifts in the relative abundance of individual bacterial families. During both periods *Rhodobacteraceae* and *Flavobacteriaceae* showed some fluctuation in abundance but remained the dominant families, and the *Halomonadaceae* increased in abundance in the eastern regions of the estuary, generally increasing with proximity to the marine conditions at the harbor mouth. In contrast, *Microbacteriaceae* and *Acidimicrobiales* OCS155 were often relatively more abundant in the western regions of the estuary (Figure 6). Combined, the *Microbacteriaceae, Halomonadaceae, Acidimicrobiales* OCS155 and *Flavobacteriaceae* accounted for 13.4% of the spatial dissimilarity between the western, upper river sites and the eastern, marine sites in February, when the estuary was most heterogeneous (Supplementary Material Table 4). In addition to being key drivers of dissimilarity using SIMPER, the differential abundance of these groups between regions of the estuary were supported by two-group statistical analyses using STAMP (Supplementary Information Figure 10).

In addition to the broad spatial and temporal trends in bacterial community composition observed here, localized sites, where hotspots in nutrient concentrations or decreased DO levels occurred (e.g., MH1, LPR1, LC1 and LC2), often hosted taxonomically discrete microbial assemblages. For example, despite being in close proximity, the Lane Cove sites LC1 and LC3, hosted substantially different microbial communities, with LC1 having a much higher relative abundance of *Acidomicrobiales* C111 and OCS155 compared to LC3. These fine-scale shifts in community characteristics are likely explained by differences in the chemical characteristics of these two sites, with LC1 characterized by lower DO and pH and higher nutrient concentrations than LC3.

**Figure 7:**
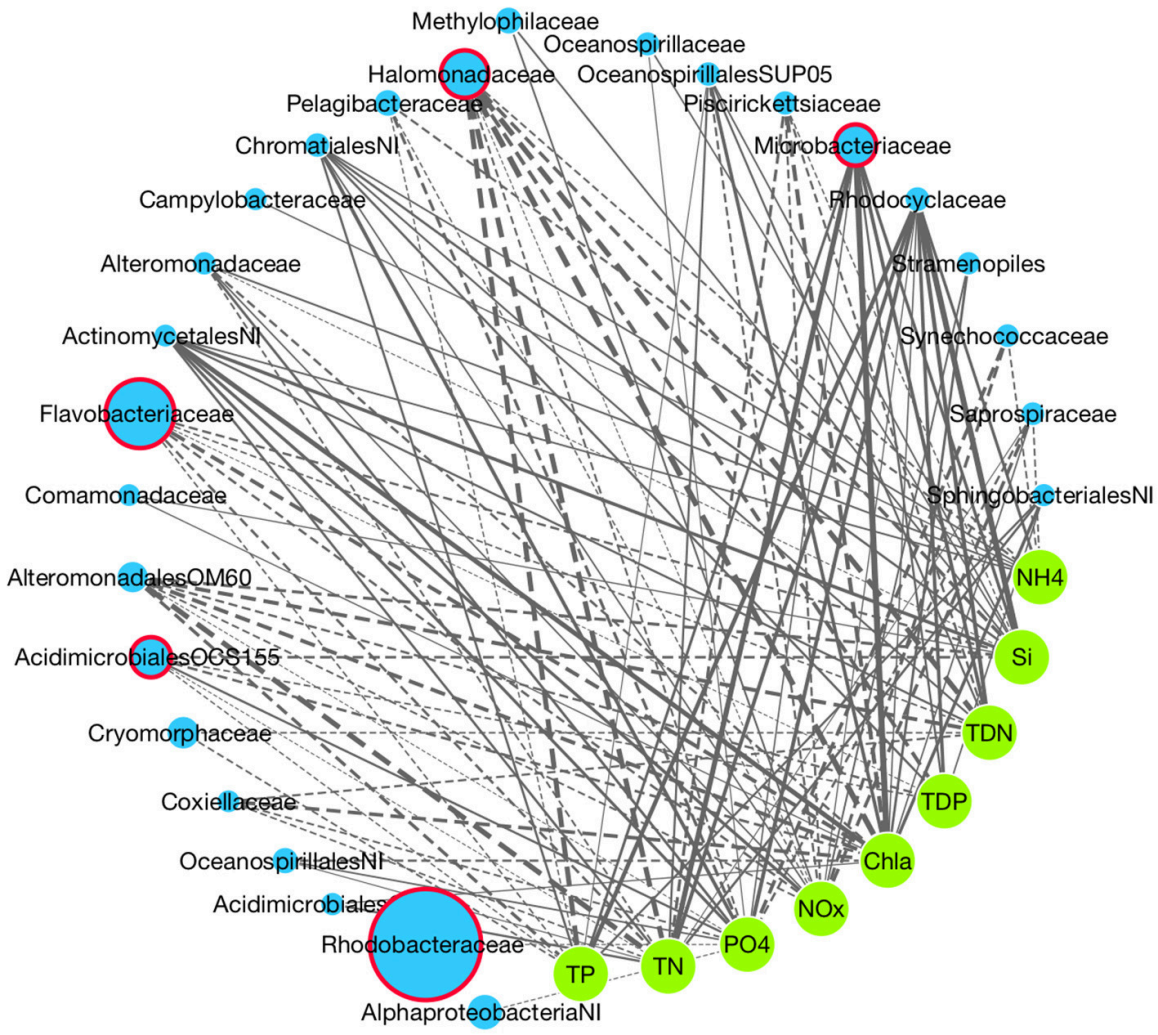
**Network analysis of associations between taxon abundances (family level) and nutrient concentration**. Blue nodes indicate taxa with the width of the node proportional to the taxon's abundance. Green nodes indicate nutrients. Positive interactions are solid lines and negative are dashed, with the edge width proportional to the strength of the relationship (MIC score). Red node borders indicate top drivers identified using SIMPER analysis.

We used network analysis to identify statistical links between the relative abundance of specific bacterial taxa and environmental conditions (Figure 7). In the overall network, which was filtered to show only significant (*P* < 0.05) relationships between taxonomic groups and nutrients, there were 115 correlations with the nutrients to which there were the most associations being ${\text{PO}}_{4}^{-}$ (16 taxa) and TN (15 taxa). The nutrients showing the strongest relationships were TDN and TN (average MIC score = 0.488 and 0.482 respectively). Chl a (indicative of high biomass and nutrient loading) was the most connected node (17 taxa, average MIC = 0.52). Several taxa that were identified as being key drivers of community dissimilarity using the SIMPER analysis were highly correlated to nutrient concentrations. In particular, the *Flavobacteriaceae* and *Halomonodaceae* showed negative correlations to seven and nine nutrients respectively, with the strongest being chl a, TN, TDN, and TP. *Microbacteriaceae*, which increased in abundance in the nutrient enriched western harbor was positively correlated to seven nutrients with the strongest relationships to chl a, TN and TDN.

### Spatial shifts in functional potential (shotgun metagenomes)

**Figure 8:**
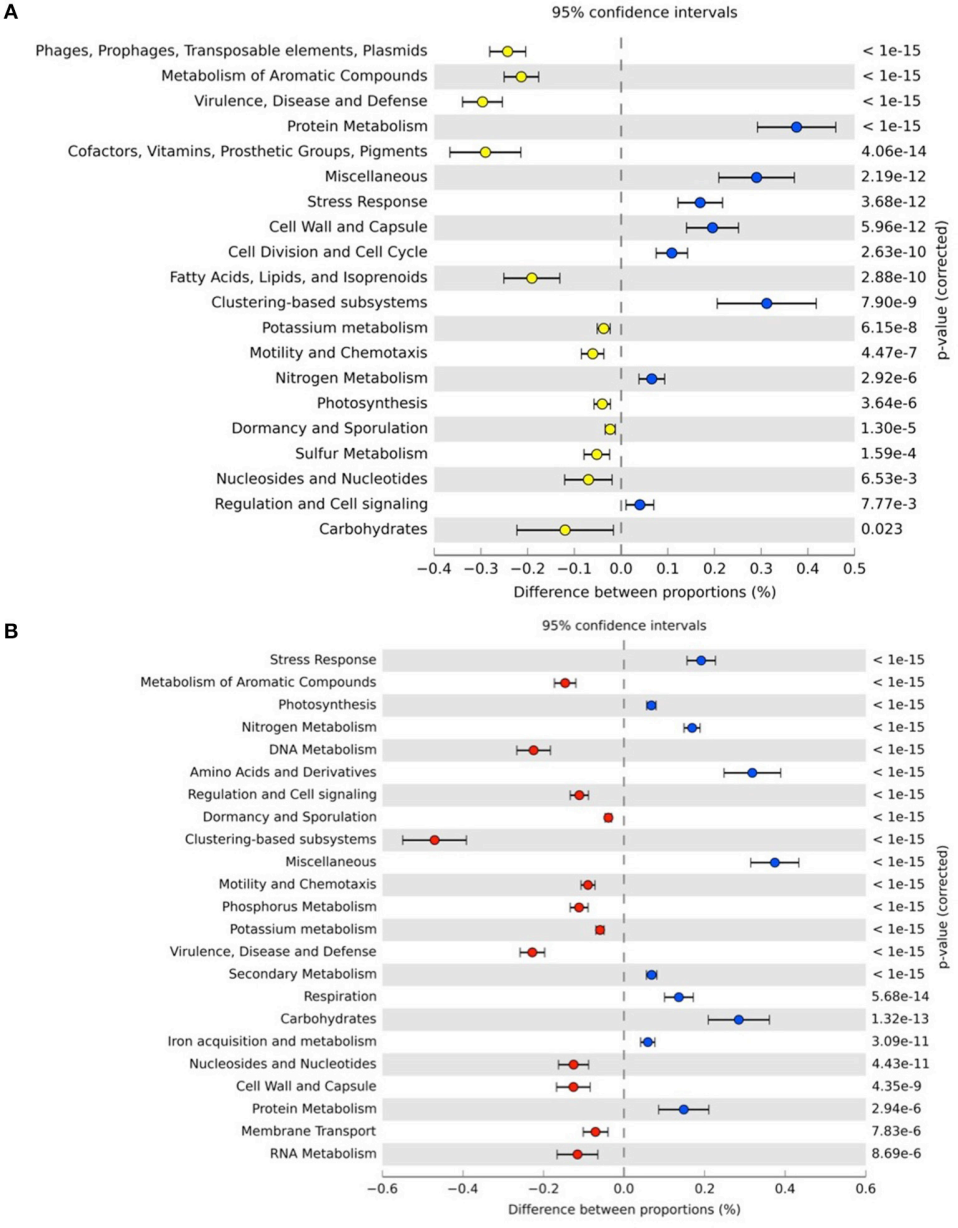
**Pairwise comparison of functional profiles between Sydney Heads (PJ7, blue circles, positive differences between proportions on right-hand side of plot) and (A) Blackwattle Bay (PJ3; yellow circles) (B) Homebush Bay (P3; red circles)**. Corrected *p*-value is determined using Fisher's exact test with a Benjamini FDR multiple test correction. Pathways are level one of SEED metabolic hierarchy.

**Figure 9:**
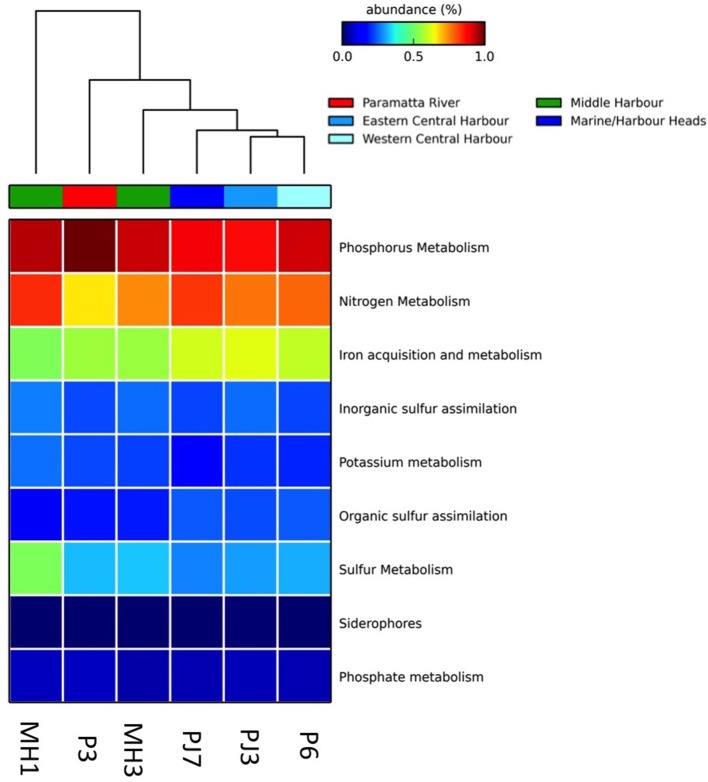
**Heatmap displaying relative abundance of functional pathways involved in nutrient cycling (level one of the SEED hierarchy)**. Dendrogram clustering represents the Bray-Curtis similarity of profile.

To investigate how spatial heterogeneity in environmental parameters influenced the functional capacity of microbial assemblages inhabiting the Sydney Harbor estuary, we conducted a metagenomic survey of six representative sites in the Harbor. At the most coarsely defined level of metabolic processes (Level 1 in the SEED hierarchy), core “house-keeping” functions, including genes encoding carbohydrates, protein metabolism, amino acid and derivatives, cofactors, and RNA metabolism were dominant (representing over 60% of sequences) across all metagenomes (Supplementary Material Figure 2). To statistically compare differences in metagenomes between different habitats within Sydney Harbor we compared the functional profile of the representative marine sample (Marine/Harbor Heads) (PJ7) against the west and river metagenomes (Eastern-central Harbor, PJ3, and Parramatta River, P3; Figure 8). Metagenomes from a western more industrialized, region of Sydney Harbor (PJ3) displayed an overrepresentation of genes involved in lateral gene transfer, degradation of toxic aromatic compounds, and virulence and disease (Figure 8A) in addition to cofactors, vitamins and pigments fatty acids/lipids and sulfur metabolism (*q* < 0.05). Relative to the marine sample (PJ7), the Parramatta river sample (P3) was over-represented in genes for aromatic compound degradation, virulence, and disease and phosphorous metabolism (*q* < 0.05) (Figure 8B). Generally, the metagenomes located further from the estuary mouth had a higher contribution of genes involved in the sulfur and phosphorus metabolism, virulence, disease and defense, and degradation of aromatic compounds (Supplementary Material Figure 2; Figure 9).

Within the context of inorganic nutrient metabolism, there were several differences observed between the different regions within the Sydney Harbor estuary. Relative to the other sites, the western-most Parramatta River metagenome (site P3) was characterized by an over-representation of genes associated with phosphorus metabolism, but interestingly an under-representation of genes involved in nitrogen metabolism (Figure 9). The Middle Harbor site (MH1) was characterized by a higher abundance of nitrogen metabolism genes (Figure 9) including nitrate and nitrite ammonification genes, as well as denitrification genes and dissimilatory nitrite reductase (Supplementary Material Figure 3, *q* < 0.05). The west and upper-river metagenomes (more eutrophic sites) displayed an over-representation of genes involved in phosphorus metabolism generally (Figure 9) including “P uptake by cyanobacteria” and “high affinity phosphate transporters” (Supplementary Material Figure 4, *q* < 0.05). The Middle Harbor metagenome (MH1), which was characterized by low DO levels, displayed an over-representation of genes involved in sulfur metabolism (Figure 9), in particular those involved in “sulfate reduction associated complexes,” “inorganic sulfur assimilation,” and “sulfur oxidation” (Supplementary Material Figure 5, *q* < 0.05). Additionally, genes associated with “phages and prophages” were higher at Middle Harbor (MH1) and Eastern-Central Harbor (PJ3), while Middle Harbor (MH1 and MH3) revealed a higher number of genes involved in “virulence, disease, and defense,” which were largely made up of antibiotic resistance pathways (Supplementary Material Figure 6).

## Discussion

By combining measurements of physicochemical conditions with assessments of patterns in microbial diversity and functional potential, we have provided a first insight into the microbial ecology of the Sydney Harbor estuary. Overall, abiotic variables and nutrient concentrations displayed significant differences between the high-rainfall period in February and the low rainfall period in September. During February we observed a higher occurrence of localized hotspots of nutrient concentrations and decreased DO, particularly within the western and up-river regions of the estuary. These patterns are indicative of localized eutrophication potentially related to point source, storm-water related inputs of nutrients. This localized variability overlayed larger scale spatial gradients in parameters including salinity and nutrients, which are reflective of both the natural environmental gradients expected within estuaries and the higher levels of urbanization and industrialization in the western regions of the estuary. The spatial heterogeneity of nutrient concentrations observed here is consistent with previous observations in Sydney Harbor that described a general trend of higher nutrient concentrations toward the western (inland) end of the harbor (Birch et al., 1999), with an enhanced influence of tidal flushing at the marine end of the system (Hedge et al., 2014).

Increased environmental heterogeneity of this system following periods of heavy rain has previously been observed (Lee et al., 2011), where at moderate rainfall levels nutrients accumulate near to input sources (Birch et al., 2010). This patchiness was evident in the nutrient distributions observed here, which were heterogeneous during both sampling periods, but in particular during February. Freshwater inflows deliver nutrients, suspended solids and contamination into the Sydney Harbor (Birch and Rochford, 2010) with hydrological models suggesting that sewage contributes over 50% of TN and TP to the estuary (Birch et al., 2010) in addition to nutrients derived from fertilizer use and anthropogenic hydrology modification (Hedge et al., 2014). The spatiotemporal differences in the magnitude of these inputs were a likely driver of many of the shifts in the composition of microbial communities observed in this study. Additionally, the direct input of microorganisms from sources such as sewage and wastewater treatment could directly influence the community composition of the system. High concentrations of microorganisms persist in wastewater systems (Vandewalle et al., 2012; Liu et al., 2015b) and have been shown to influence the function and nutrient cycles of aquatic habitats (Mußmann et al., 2013). Fecal coliforms are known to be present in Sydney Harbor (Hose et al., 2005) and wastewater is thought to be a direct source of microbial contamination into the estuary (Hedge et al., 2014).

Our data indicated that bacterial assemblages inhabiting the Sydney Harbor estuary exhibit substantial shifts in composition in both space and time, which can be explained by patterns in physical conditions and nutrient concentrations. The clear partitioning of community beta-diversity and shifts in relative abundance of taxa between a period of high-rainfall (February) and low-rainfall (September) are potentially linked to allochtonous inputs driven by increased inflow from rainfall. Temporal shifts and seasonality in microbial assemblages are well established in aquatic habitats (Fuhrman et al., 2006, 2015) and have been linked to increased anthropogenic nutrient loads (Perryman et al., 2011). In Sydney Harbor specifically, increased stormwater runoff during wet periods has been shown to drive variability in coliform and enterococci concentrations, particularly in the less often flushed western reaches of the system (Hose et al., 2005). Here we extend upon these previous culture-dependent observations to demonstrate that broad community-level shifts occur in both space and time.

Temperature, salinity, pH, dissolved DO and phosphate were identified as key drivers of the spatiotemporal dissimilarity between microbial assemblages inhabiting different regions of the Sydney Harbor estuary. In this study we have considered salinity, temperature, and pH as naturally varying parameters, and increasing nutrient concentrations and low DO levels as potentially anthropogenic influences in addition to obvious anthropogenic inputs such as hydrocarbons and chemical toxins. Salinity and temperature are common natural drivers of microbial biogeography in estuaries, with the microbial assemblages inhabiting the typically warmer, low salinity upper regions of estuaries often displaying markedly different community characteristics to those observed in the often cooler, marine conditions near to estuary mouths (Schultz et al., 2003; Crump et al., 2004; Kirchman et al., 2005; Campbell and Kirchman, 2013). Whilst some nutrient levels are the result of natural processes, anthropogenic sources are responsible for significant proportions of nutrient input in this system (Birch et al., 2010; Hedge et al., 2014). Both natural gradients in organic and inorganic nutrients and point source inputs of high concentrations of nutrients associated with stormwater, agricultural run-off and sewage create heterogeneity in nutrient distributions within estuaries (Paerl, 2006; Birch et al., 2010; Liu et al., 2015a). In particular, phosphorous, and phosphates are common contaminants in urban and agricultural runoff and lead to eutrophication in aquatic systems; resulting in community shifts, reduced oxygen levels and potentially blooms of harmful organisms (Correll, 1998; Anderson et al., 2002; Paerl et al., 2003; González-Ortegón and Drake, 2012; Carney et al., 2015). We found that within Sydney Harbor, phosphate was a principle driver of differences among microbial assemblages and found that localized hotspots of phosphate concentration often coincided with the occurrence of microbial assemblages with differing community characteristics to those observed throughout other regions of the harbor. Often increased eutrophication drives decreases in oxygen availability in aquatic systems (Paerl, 2006; González-Ortegón and Drake, 2012) and dissolved oxygen is a major factor in structuring aquatic microbial communities (Crump et al., 2007; Wright et al., 2012; Laas et al., 2015), particularly those inhabiting the freshwater zones of estuaries (Liu et al., 2015a) as was observed here. Thus, shifts in microbial community composition with nutrients may also correspond with anoxic hotspots and peaks in anaerobic bacteria such as the Purple Sulfur Bacteria (Chromatiales), as was observed at several up-river sites in our dataset.

To further elucidate the interaction between specific bacterial taxa and measured environmental parameters we applied a network analysis approach. Generally nutrients were highly correlated to the abundance of specific taxa and both phosphate (${\text{PO}}_{4}^{-}$) and Total Nitrogen (TN) were the most highly connected nutrients further highlighting their role in structuring the bacterioplankton community. The *Microbacteriaceae* and a marine clade of Acidimicrobiale*s*, previously shown to exhibit strong temporal dynamics in aquatic systems (Needham et al., 2013), both showed strong positive associations with phosphate and other nutrients. Both taxa were among the top drivers of community dissimilarity identified using SIMPER analysis, which when linked to the correlations observed using network analysis confirm that inorganic nutrient concentrations are a principle driver of microbial community dynamics within the Sydney Harbor estuary. However, other major contributors to community dissimilarity such as *Halomonodaceae* and *Flavobacteriaceae* were negatively correlated to nutrient concentrations, as were *Pelagibacter* and *Synechococcu*s. These taxa are all common in marine habitats (Arahal and Ventosa, 2006; Gómez-Pereira et al., 2010; Brown et al., 2012; Mazard et al., 2012) and showed higher abundances in the eastern seaward regions of the estuary, reflecting the preferences of these organisms for marine salinity and lower nutrient concentrations. Interestingly, the most abundant bacterial family across Sydney Harbor, the *Rhodobacteraceae*, was not significantly associated with nutrient concentrations, suggesting the distribution of these bacteria is controlled by other factors such as salinity and temperature, or that there is heterogeneity of nutrient acquisition strategies at finer phylogenetic levels within this group. This family belongs to the order Rhodobacterales, which is highly abundant in the marine environment (Gilbert et al., 2010) and has previously been found to increase in abundance at the marine end of estuaries (Campbell and Kirchman, 2013; Liu et al., 2015a).

Spatial shifts in the metabolic potential of the bacterial communities inhabiting the Sydney Harbor estuary also indicated the links between environmental variables and microbial function in the estuary. This is consistent with other metagenomic surveys in aquatic habitats (Rusch et al., 2007; DeLong, 2009; Gilbert et al., 2010) and along salinity gradients (Jeffries et al., 2012). Over-representation of metabolic pathways involved in the degradation of aromatic compounds (hydrocarbons), degradation of toxic compounds, and virulence observed in metagenomes from the more industrialized sites in the western region of the harbor, provide further evidence for the influence of anthropogenic activity on microbial function.

Strong patterns in pathways involved in nutrient cycling corresponded to gradients in nutrient concentration and hotspots of substrates and anoxia. The observation that pathways involved in phosphorous utilization were higher in the metagenomes collected at the more eutrophic inland and river sites is congruent with the high concentrations of phosphate and total phosphorous in these locations. The increase in metabolic pathways associated with phosphorous and nitrogen cycling in specific sites indicated that Sydney Harbor microbial communities exhibit functional responses to allochtonous pulses of these chemicals, potentially influencing overall nutrient flux and aquatic health. Similarly a strong peak in the abundance of genes involved in sulfur utilization occurred in the metagenome from middle harbor (MH1), which also corresponded to a peak in the abundance of the Chromatiales. This site was characterized by relatively stagnant conditions, highly anoxic and high nutrient concentrations. The metagenome from this site also had an over-representation of genes involved in sulfate reduction, relative to the other environments, which is also consistent with a low oxygen, substrate rich habitat. This trend of increasing nutrient metabolism gene content in eutrophic conditions may only be relevant for some abundant lineages within taxonomic groups, as in some cases oligotrophic conditions have been shown to increase the diversity and significance of pathways such as those involved in phosphate utilization in the open ocean (Martiny et al., 2009; Temperton et al., 2011).

Taken together these metagenomic results highlight the influence of anthropogenic inputs from effluent, industry, and agriculture on key microbial functions such as hydrocarbon degradation (industry) and nutrient cycling (run-off and agriculture) respectively. In particular, genes involved in the nitrogen and phosphorous cycling are potential influenced by eutrophication as a result of fertilizer use in agriculture and in high nutrient loadings in sewage. Genes related to hydrocarbon degradation and virulence are potentially related to industry and run-off from human waste respectively. Together with the 16S rRNA amplicon data, these results are consistent with studies in other estuarine environments that have linked the distributional dynamics of bacterial communities to nutrient gradients (Crump et al., 2004; Jeffries et al., 2012; Fortunato et al., 2013; Liu et al., 2015a) and allochtonous nutrient pulses (Carney et al., 2015). The links between nutrient enrichment, likely derived from stormwater and sewage inputs (Birch et al., 2010; Hedge et al., 2014), and microbial biogeography within Sydney Harbor highlight the influence of anthropogenic forces on defining the microbial ecology of urban estuaries. We acknowledge that it is not always possible to delineate between the mechanism behind physicochemical heterogeneity and that nutrient variability could be simultaneously driven by natural and anthropogenic influences. Based on previous literature however (e.g., Birch et al., 2010; Hedge et al., 2014) we are confident that large amounts of the input of nutrients to this system were derived from anthropogenic sources. Due to the multiple sources of impact and nutrient input in the Sydney Harbor estuary, particularly within the western regions of the ecosystem, it is not possible to discriminate single sources (e.g., industry, agriculture, and sewage over-flow) of the eutrophication underpinning the shifts in the microbial community. Nonetheless, the cumulative impact of a variety of inputs in the western region of the Sydney Harbor estuary have clearly led to elevated nutrient levels and associated shifts in the composition and function of the microbial communities.

## Conclusion

By combining physicochemical, taxonomic and metabolic datasets, this study has demonstrated the influence of both natural (e.g., temperature and salinity) and anthropogenically enhanced (e.g., high nutrients and low DO) environmental variability on defining microbial phylogenetic and functional biogeography in an urbanized estuary. As the first detailed survey of microbial diversity in Sydney Harbor, the most heavily populated region of coastline in Australia, this study revealed the highly dynamic spatiotemporal patterns in microbial communities within this habitat, which were linked to environmental heterogeneity driven by the natural physicochemical gradients, the influence of a rainfall event and allochtonous nutrient inputs. The apparent links between potentially anthropogenically derived factors and microbial biogeography highlights the need for future studies which directly correlate individual microbial taxa and functional pathways to anthropogenic sources of input under low and high freshwater flows. This will lead to a better-understanding of the factors which underlie shifts in microbial composition and function in urbanized aquatic systems, and the consequential effects on aquatic and human health.

### Conflict of interest statement

The authors declare that the research was conducted in the absence of any commercial or financial relationships that could be construed as a potential conflict of interest.

## References

[B1] Abell G. C. J. Ross D. J. Keane J. Holmes B. H. Robert S. S. Keough M. J.. (2014). Niche differentiation of ammonia-oxidising archaea (AOA) and bacteria (AOB) in response to paper and pulp mill effluent. Microb. Ecol. 67, 758–768. 10.1007/s00248-014-0376-7 24563191

[B2] Abell G. C. J. Ross D. J. Keane J. P. Oakes J. M. Eyre B. D. Robert S. S. (2013). Nitrifying and denitrifying microbial communities and their relationship to nutrient fluxes and sediment geochemistry in the Derwent Estuary, Tasmania. Aquat. Microb. Ecol. 70, 63–75. 10.3354/ame01642

[B3] Altschul S. F. Gish W. Miller W. Myers E. W. Lipman D. J. (1990). Basic local alignment search tool. J. Mol. Biol. 215, 403–410. 10.1016/S0022-2836(05)80360-2 2231712

[B4] Anderson D. M. Glibert P. M. Burkholder J. M. (2002). Harmful algal blooms and eutrophication: nutrient sources, composition, and consequences. Estuaries 25, 704–726. 10.1007/BF02804901

[B5] Anderson M. Gorley R. Clarke K. (2008). Permanova+ For Primer: A Guide to Software and Statistical Methods. Plymouth: Primer-e.

[B6] American Public Health Association (APHA) (2005). Standard Methods for the Examination of water and Wastewater. Washington, DC: APHA.

[B7] Arahal D. R. Ventosa A. (2006). The family Halomonadaceae, in The Prokaryotes, eds Dworkin M. Falkow S. Rosenberg E. Schleifer K. H. Stackebrandt E. (New York, NY: Springer), 811–835.

[B8] Beck H. J. Birch G. F. (2012). Metals, nutrients and total suspended solids discharged during different flow conditions in highly urbanised catchments. Environ. Monit. Assess. 184, 637–653. 10.1007/s10661-011-1992-z 21448629

[B9] Benjamini Y. Hochberg Y. (1995). Controlling the false discovery rate: a practical and powerful approach to multiple testing. J. R. Stat. Soc. B 57, 289–300.

[B10] Birch G. F. Cruickshank B. Davis B. (2010). Modelling nutrient loads to Sydney estuary (Australia). Environ. Monit. Assess. 167, 333–348. 10.1007/s10661-009-1053-z 19568942

[B11] Birch G. F. Eyre B. Taylor S. E. (1999). The distribution of nutrients in bottom sediments of Port Jackson (Sydney Harbour), Australia. Mar. Pollut. Bull. 38, 1247–1251. 10.1016/S0025-326X(99)00184-8

[B12] Birch G. F. Rochford L. (2010). Stormwater metal loading to a well-mixed/stratified estuary (Sydney Estuary, Australia) and management implications. Environ. Monit. Assess. 169, 531–551. 10.1007/s10661-009-1195-z 19859822

[B13] Bouvier T. C. Del Giorgio P. A. (2002). Compositional changes in free-living bacterial communities along a salinity gradient in two temperate estuaries. Limnol. Oceanogr. 47, 453–470. 10.4319/lo.2002.47.2.0453

[B14] Breitburg D. L. Crump B. C. Dabiri J. O. Gallegos C. L. (2010). Ecosystem engineers in the pelagic realm: alteration of habitat by species ranging from microbes to jellyfish. Integr. Comp. Biol. 50, 188–200. 10.1093/icb/icq051 21558198

[B15] Brown M. V. Lauro F. M. DeMaere M. Z. Muir L. Wilkins D. Thomas T.. (2012). Global biogeography of SAR11 marine bacteria. Mol. Syst. Biol. 8, 595. 10.1038/msb.2012.28 22806143 PMC3421443

[B16] Campbell B. J. Kirchman D. L. (2013). Bacterial diversity, community structure and potential growth rates along an estuarine salinity gradient. ISME J. 7, 210–220. 10.1038/ismej.2012.93 22895159 PMC3526181

[B17] Caporaso J. G. Bittinger K. Bushman F. D. DeSantis T. Z. Andersen G. L. Knight R. (2010a). PyNAST: a flexible tool for aligning sequences to a template alignment. Bioinformatics 26, 266–267. 10.1093/bioinformatics/btp636 19914921 PMC2804299

[B18] Caporaso J. G. Kuczynski J. Stombaugh J. Bittinger K. Bushman F. D. Costello E. K.. (2010b). QIIME allows analysis of high-throughput community sequencing data. Nat. Methods 7, 335–336. 10.1038/nmeth.f.303 20383131 PMC3156573

[B19] Carney R. L. Mitrovic S. M. Jeffries T. Westhorpe D. Curlevski N. Seymour J. R. (2015). River bacterioplankton community responses to a high inflow event. Aquat. Microb. Ecol. 75, 187–205. 10.3354/ame01758

[B20] Chen L. X. Hu M. Huang L. N. Hua Z. S. Kuang J. L. Li S. J.. (2015). Comparative metagenomic and metatranscriptomic analyses of microbial communities in acid mine drainage. ISME J. 9, 1579–1592. 10.1038/ismej.2014.245 25535937 PMC4478699

[B21] Clarke K. R. (1993). Non-parametric multivariate analyses of changes in community structure. Aust. J. Ecol. 18, 117–117. 10.1111/j.1442-9993.1993.tb00438.x

[B22] Clarke K. R. Gorley R. N. (2006). User Manual/Tutorial. Plymouth: PRIMER-E Ltd.

[B23] Cole J. J. (1999). Aquatic microbiology for ecosystem scientists: new and recycled paradigms in ecological microbiology. Ecosystems 2, 215–225. 10.1007/s100219900069

[B24] Correll D. L. (1998). The role of phosphorus in the eutrophication of receiving waters: a review. J. Environ. Qual. 27, 261–266. 10.2134/jeq1998.00472425002700020004x

[B25] Crump B. C. Hopkinson C. S. Sogin M. L. Hobbie J. E. (2004). Microbial biogeography along an estuarine salinity gradient: combined influences of bacterial growth and residence time. Appl. Environ. Microbiol. 70, 1494–1505. 10.1128/AEM.70.3.1494-1505.2004 15006771 PMC365029

[B26] Crump B. C. Peranteau C. Beckingham B. Cornwell J. C. (2007). Respiratory succession and community succession of bacterioplankton in seasonally anoxic estuarine waters. Appl. Environ. Microbiol. 73, 6802–6810. 10.1128/AEM.00648-07 17766441 PMC2074974

[B27] Das P. Marchesiello P. Middleton J. H. (2000). Numerical modelling of tide-induced residual circulation in Sydney Harbour. Mar. Freshw. Res. 51, 97–112. 10.1071/MF97177

[B28] DeLong E. F. (2009). The microbial ocean from genomes to biomes. Nature 459, 200–206. 10.1038/nature08059 19444206

[B29] DeSantis T. Z. Hugenholtz P. Larsen N. Rojas M. Brodie E. L. Keller K.. (2006). Greengenes, a chimera-checked 16S rRNA gene database and workbench compatible with ARB. Appl. Environ. Microbiol. 72, 5069–5072. 10.1128/AEM.03006-05 16820507 PMC1489311

[B30] Diaz R. J. Rosenberg R. (2008). Spreading dead zones and consequences for marine ecosystems. Science 321, 926–929. 10.1126/science.1156401 18703733

[B31] Edgar R. C. (2010). Search and clustering orders of magnitude faster than BLAST. Bioinformatics 26, 2460–2461. 10.1093/bioinformatics/btq461 20709691

[B32] Engelbrektson A. Kunin V. Wrighton K. C. Zvenigorodsky N. Chen F. Ochman H.. (2010). Experimental factors affecting PCR-based estimates of microbial species richness and evenness. ISME J. 4, 642–647. 10.1038/ismej.2009.153 20090784

[B33] Eyre B. D. (2000). Regional evaluation of nutrient transformation and phytoplankton growth in nine river-dominated sub-tropical east Australian estuaries. Mar. Ecol. Prog. Ser. 205, 61–83. 10.3354/meps205061

[B34] Fortunato C. S. Eiler A. Herfort L. Needoba J. A. Peterson T. D. Crump B. C. (2013). Determining indicator taxa across spatial and seasonal gradients in the Columbia River coastal margin. ISME J. 7, 1899–1911. 10.1038/ismej.2013.79 23719153 PMC3965310

[B35] Fortunato C. S. Herfort L. Zuber P. Baptista A. M. Crump B. C. (2012). Spatial variability overwhelms seasonal patterns in bacterioplankton communities across a river to ocean gradient. ISME J. 6, 554–563. 10.1038/ismej.2011.135 22011718 PMC3280145

[B36] Fuhrman J. A. Cram J. A. Needham D. M. (2015). Marine microbial community dynamics and their ecological interpretation. Nat. Rev. Microbiol. 13, 133–146. 10.1038/nrmicro3417 25659323

[B37] Fuhrman J. A. Hewson I. Schwalbach M. S. Steele J. A. Brown M. V. Naeem S. (2006). Annually reoccurring bacterial communities are predictable from ocean conditions. Proc. Nat. Acad. Sci. U.S.A. 103, 13104–13109. 10.1073/pnas.0602399103 16938845 PMC1559760

[B38] Gibbons S. M. Caporaso J. G. Pirrung M. Field D. Knight R. Gilbert J. A. (2013). Evidence for a persistent microbial seed bank throughout the global ocean. Proc. Nat. Acad. Sci. U.S.A. 110, 4651–4655. 10.1073/pnas.1217767110 23487761 PMC3607043

[B39] Gilbert J. A. Field D. Swift P. Thomas S. Cummings D. Temperton B.. (2010). The taxonomic and functional diversity of microbes at a temperate coastal site: a ‘multi-omic’study of seasonal and diel temporal variation. PLoS ONE 5:e15545. 10.1371/journal.pone.0015545 21124740 PMC2993967

[B40] Gillan D. C. Danis B. Pernet P. Joly G. Dubois P. (2005). Structure of sediment-associated microbial communities along a heavy-metal contamination gradient in the marine environment. Appl. Environ. Microbiol. 71, 679–690. 10.1128/AEM.71.2.679-690.2005 15691917 PMC546797

[B41] Glass E. M. Wilkening J. Wilke A. Antonopoulos D. Meyer F. (2010). Using the metagenomics RAST server (MG-RAST) for analyzing shotgun metagenomes. Cold Spring Harb. Protoc. 2010:prot5368. 10.1101/pdb.prot5368 20150127

[B42] Gomez-Alvarez V. Teal T. K. Schmidt T. M. (2009). Systematic artifacts in metagenomes from complex microbial communities. ISME J. 3, 1314–1317. 10.1038/ismej.2009.72 19587772

[B43] Gómez-Pereira P. R. Fuchs B. M. Alonso C. Oliver M. J. van Beusekom J. E. Amann R. (2010). Distinct flavobacterial communities in contrasting water masses of the North Atlantic Ocean. ISME J. 4, 472–487. 10.1038/ismej.2009.142 20054356

[B44] González-Ortegón E. Drake P. (2012). Effects of freshwater inputs on the lower trophic levels of a temperate estuary: physical, physiological or trophic forcing? Aquat. Sci. 74, 455–469. 10.1007/s00027-011-0240-525343621

[B45] Gregoracci G. B. Nascimento J. R. Cabral A. S. Paranhos R. Valentin J. L. Thompson C. C.. (2012). Structuring of bacterioplankton diversity in a large tropical bay. PLoS ONE 7:e31408. 10.1371/journal.pone.0031408 22363639 PMC3283626

[B46] Haas B. J. Gevers D. Earl A. M. Feldgarden M. Ward D. V. Giannoukos G.. (2011). Chimeric 16S rRNA sequence formation and detection in Sanger and 454-pyrosequenced PCR amplicons. Genome Res. 21, 494–504. 10.1101/gr.112730.110 21212162 PMC3044863

[B47] Hatje V. Apte S. C. Hales L. T. Birch G. F. (2003). Dissolved trace metal distributions in Port Jackson estuary (Sydney Harbour), Australia. Mar. Pollut. Bull. 46, 719–730. 10.1016/S0025-326X(03)00061-4 12787580

[B48] Head I. M. Jones D. M. Röling W. F. M. (2006). Marine microorganisms make a meal of oil. Nat. Rev. Microbiol. 4, 173–182. 10.1038/nrmicro1348 16489346

[B49] Hedge L. Johnston E. Ahyong S. Birch G. Booth D. Creese B. (2014). Sydney Harbour: A Systematic Review of the Science. Sydney: The Sydney Institute of Marine Science.

[B50] Hose G. C. Gordon G. McCullough F. E. Pulver N. Murray B. R. (2005). Spatial and rainfall related patterns of bacterial contamination in Sydney Harbour estuary. J. Water Health 3, 349–358. 10.2166/wh.2005.060 16459841

[B51] Hsieh J. L. Fries J. S. Noble R. T. (2007). Vibrio and phytoplankton dynamics during the summer of 2004 in a eutrophying estuary. Ecolo. Appl. 17, S102–S109. 10.1890/05-1274.1

[B52] Jeffries T. C. Seymour J. R. Newton K. Smith R. J. Seuront L. Mitchell J. G. (2012). Increases in the abundance of microbial genes encoding halotolerance and photosynthesis along a sediment salinity gradient. Biogeosciences 9, 815–825. 10.5194/bg-9-815-2012

[B53] Keegan K. P. Trimble W. L. Wilkening J. Wilke A. Harrison T. D'souza M.. (2012). A platform-independent method for detecting errors in metagenomic sequencing data: drisee. PLoS Comput. Biol. 8:e1002541. 10.1371/journal.pcbi.1002541 22685393 PMC3369934

[B54] Kent W. J. (2002). BLAT—the BLAST-like alignment tool. Genome Res. 12, 656–664. 10.1101/gr.229202 11932250 PMC187518

[B55] Kirchman D. L. Dittel A. I. Malmstrom R. R. Cottrell M. T. (2005). Biogeography of major bacterial groups in the Delaware Estuary. Limnol. Oceanogr. 50, 1697–1706. 10.4319/lo.2005.50.5.1697

[B56] Laas P. Simm J. Lips I. Lips U. Kisand V. Metsis M. (2015). Redox-specialized bacterioplankton metacommunity in a temperate estuary. PLoS ONE 10:e0122304. 10.1371/journal.pone.0122304 25860812 PMC4393233

[B57] Lee S. B. Birch G. F. Lemckert C. J. (2011). Field and modelling investigations of fresh-water plume behaviour in response to infrequent high-precipitation events, Sydney Estuary, Australia. Estuar. Coast. Shelf Sci. 92, 389–402. 10.1016/j.ecss.2011.01.013

[B58] Line D. E. White N. M. (2007). Effects of development on runoff and pollutant export. Water Environ. Res. 79, 185–190. 10.2175/106143006X111736 17370844

[B59] Liu J. Fu B. Yang H. Zhao M. He B. Zhang X.-H. (2015a). Phylogenetic shifts of bacterioplankton community composition along the Pearl Estuary: the potential impact of hypoxia and nutrients. Front. Microbiol. 6:64. 10.3389/fmicb.2015.00064 25713564 PMC4322608

[B60] Liu Y. Dong Q. Shi H. (2015b). Distribution and population structure characteristics of microorganisms in urban sewage system. Appl. Microbiol. Biotechnol. 99, 7723–7734. 10.1007/s00253-015-6661-7 25981998

[B61] Lozupone C. Knight R. (2005). UniFrac: a new phylogenetic method for comparing microbial communities. Appl. Environ. Microbiol. 71, 8228–8235. 10.1128/AEM.71.12.8228-8235.2005 16332807 PMC1317376

[B62] Maher W. Krikowa F. Wruck D. Louie H. Nguyen T. Huang W. Y. (2002). Determination of total phosphorus and nitrogen in turbid waters by oxidation with alkaline potassium peroxodisulfate and low pressure microwave digestion, autoclave heating or the use of closed vessels in a hot water bath: comparison with Kjeldahl digestion. Anal. Chim. Acta 463, 283–293. 10.1016/S0003-2670(02)00346-X

[B63] Martiny A. C. Huang Y. Li W. (2009). Occurrence of phosphate acquisition genes in Prochlorococcus cells from different ocean regions. Environ. Microbiol. 11, 1340–1347. 10.1111/j.1462-2920.2009.01860.x 19187282

[B64] Mazard S. Ostrowski M. Partensky F. Scanlan D. J. (2012). Multi-locus sequence analysis, taxonomic resolution and biogeography of marine *Synechococcus*. Environ. Microbiol. 14, 372–386. 10.1111/j.1462-2920.2011.02514.x 21651684

[B65] McCready S. Birch G. F. Long E. R. (2006). Metallic and organic contaminants in sediments of Sydney Harbour, Australia and vicinity—a chemical dataset for evaluating sediment quality guidelines. Environ. Int. 32, 455–465. 10.1016/j.envint.2005.10.006 16337000

[B66] McCready S. Slee D. J. Birch G. F. Taylor S. E. (2000). The distribution of polycyclic aromatic hydrocarbons in surficial sediments of Sydney Harbour, Australia. Mar. Pollut. Bull. 40, 999–1006. 10.1016/S0025-326X(00)00044-8

[B67] McDonald D. Price M. N. Goodrich J. Nawrocki E. P. Desantis T. Z. Probst A.. (2012). An improved Greengenes taxonomy with explicit ranks for ecological and evolutionary analyses of bacteria and archaea. ISME J. 6, 610–618. 10.1038/ismej.2011.139 22134646 PMC3280142

[B68] Mendes L. W. Kuramae E. E. Navarrete A. A. van Veen J. A. Tsai S. M. (2014). Taxonomical and functional microbial community selection in soybean rhizosphere. ISME J. 8, 1577–1587. 10.1038/ismej.2014.17 24553468 PMC4817605

[B69] Meyer F. Paarmann D. D'souza M. Olson R. Glass E. M. Kubal M.. (2008). The metagenomics RAST server–a public resource for the automatic phylogenetic and functional analysis of metagenomes. BMC Bioinformatics 9:386. 10.1186/1471-2105-9-386 18803844 PMC2563014

[B70] Mußmann M. Ribot M. von Schiller D. Merbt S. N. Augspurger C. Karwautz C.. (2013). Colonization of freshwater biofilms by nitrifying bacteria from activated sludge. FEMS Microbiol. Ecol. 85, 104–115. 10.1111/1574-6941.12103 23461285

[B71] Needham D. M. Chow C.-E. T. Cram J. A. Sachdeva R. Parada A. Fuhrman J. A. (2013). Short-term observations of marine bacterial and viral communities: patterns, connections and resilience. ISME J. 7, 1274–1285. 10.1038/ismej.2013.19 23446831 PMC3695287

[B72] Overbeek R. Begley T. Butler R. M. Choudhuri J. V. Chuang H.-Y. Cohoon M.. (2005). The subsystems approach to genome annotation and its use in the project to annotate 1000 genomes. Nucleic Acids Res. 33, 5691–5702. 10.1093/nar/gki866 16214803 PMC1251668

[B73] Paerl H. W. (1997). Coastal eutrophication and harmful algal blooms: Importance of atmospheric deposition and groundwater as" new" nitrogen and other nutrient sources. Limnol. Oceanogr. 42, 1154–1165. 10.4319/lo.1997.42.5_part_2.1154

[B74] Paerl H. W. (2006). Assessing and managing nutrient-enhanced eutrophication in estuarine and coastal waters: interactive effects of human and climatic perturbations. Ecol. Eng. 26, 40–54. 10.1016/j.ecoleng.2005.09.006

[B75] Paerl H. W. Dyble J. Moisander P. H. Noble R. T. Piehler M. F. Pinckney J. L.. (2003). Microbial indicators of aquatic ecosystem change: current applications to eutrophication studies. FEMS Microbiol. Ecol. 46, 233–246. 10.1016/S0168-6496(03)00200-9 19719555

[B76] Parks D. H. Beiko R. G. (2010). Identifying biologically relevant differences between metagenomic communities. Bioinformatics 26, 715–721. 10.1093/bioinformatics/btq041 20130030

[B77] Parks D. H. Tyson G. W. Hugenholtz P. Beiko R. G. (2014). STAMP: statistical analysis of taxonomic and functional profiles. Bioinformatics 30, 3123–3124. 10.1093/bioinformatics/btu494 25061070 PMC4609014

[B78] Perryman S. E. Rees G. N. Walsh C. J. Grace M. R. (2011). Urban stormwater runoff drives denitrifying community composition through changes in sediment texture and carbon content. Microb. Ecol. 61, 932–940. 10.1007/s00248-011-9833-8 21384215

[B79] Price M. N. Dehal P. S. Arkin A. P. (2010). FastTree 2–approximately maximum-likelihood trees for large alignments. PLoS ONE 5:e9490. 10.1371/journal.pone.0009490 20224823 PMC2835736

[B80] Reshef D. N. Reshef Y. A. Finucane H. K. Grossman S. R. Mcvean G. Turnbaugh P. J.. (2011). Detecting novel associations in large data sets. Science 334, 1518–1524. 10.1126/science.1205438 22174245 PMC3325791

[B81] Rivals I. Personnaz L. Taing L. Potier M.-C. (2007). Enrichment or depletion of a GO category within a class of genes: which test? Bioinformatics 23, 401–407. 10.1093/bioinformatics/btl633 17182697

[B82] Rusch D. B. Halpern A. L. Sutton G. Heidelberg K. B. Williamson S. Yooseph S.. (2007). The Sorcerer II global ocean sampling expedition: northwest Atlantic through eastern tropical Pacific. PLoS Biol. 5:e77. 10.1371/journal.pbio.0050077 17355176 PMC1821060

[B83] Schultz G. E. White E. D. Ducklow H. W. (2003). Bacterioplankton dynamics in the York River estuary: primary influence of temperature and freshwater inputs. Aquat. Microb. Ecol. 30, 135–148. 10.3354/ame030135

[B84] Shannon P. Markiel A. Ozier O. Baliga N. S. Wang J. T. Ramage D.. (2003). Cytoscape: a software environment for integrated models of biomolecular interaction networks. Genome Res. 13, 2498–2504. 10.1101/gr.1239303 14597658 PMC403769

[B85] Shiah F.-K. Wu T.-H. Li K.-Y. Kao S.-J. Tseng Y.-F. Chung J.-L. (2006). Thermal effects on heterotrophic processes in a coastal ecosystem adjacent to a nuclear power plant. Mar. Ecol. Prog. Ser. 309, 55–65. 10.3354/meps309055

[B86] Smith M. W. Herfort L. Tyrol K. Suciu D. Campbell V. Crump B. C.. (2010). Seasonal changes in bacterial and archaeal gene expression patterns across salinity gradients in the Columbia River coastal margin. PLoS ONE 5:e13312. 10.1371/journal.pone.0013312 20967204 PMC2954162

[B87] Sun M. Y. Dafforn K. A. Brown M. V. Johnston E. L. (2012). Bacterial communities are sensitive indicators of contaminant stress. Mar. Pollut. Bull. 64, 1029–1038. 10.1016/j.marpolbul.2012.01.035 22385752

[B88] Sun M. Y. Dafforn K. A. Johnston E. L. Brown M. V. (2013). Core sediment bacteria drive community response to anthropogenic contamination over multiple environmental gradients. Environ. Microbiol. 15, 2517–2531. 10.1111/1462-2920.12133 23647974

[B89] Temperton B. Gilbert J. A. Quinn J. P. McGrath J. W. (2011). Novel analysis of oceanic surface water metagenomes suggests importance of polyphosphate metabolism in oligotrophic environments. PLoS ONE 6:e16499. 10.1371/journal.pone.0016499 21305044 PMC3030594

[B90] Tout J. Jeffries T. C. Petrou K. Tyson G. W. Webster N. S. Garren M.. (2015). Chemotaxis by natural populations of coral reef bacteria. ISME J. 9, 1764–1777 10.1038/ismej.2014.261 25615440 PMC4511932

[B91] Vandewalle J. L. Goetz G. W. Huse S. M. Morrison H. G. Sogin M. L. Hoffmann R. G.. (2012). Acinetobacter, Aeromonas and Trichococcus populations dominate the microbial community within urban sewer infrastructure. Environ. Microbiol. 14, 2538–2552. 10.1111/j.1462-2920.2012.02757.x 22524675 PMC3427404

[B92] Vieira R. P. Gonzalez A. M. Cardoso A. M. Oliveira D. N. Albano R. M. Clementino M. M.. (2008). Relationships between bacterial diversity and environmental variables in a tropical marine environment, Rio de Janeiro. Environ. Microbiol. 10, 189–199. 10.1111/j.1462-2920.2007.01443.x 17892478

[B93] Wilke A. Glass E. M. Bichof J. Braithwaite D. D'souza M. Gerlach W. (2014). MG-RAST. Manual for version 3.3.6, revision 9. Available online at : ftp://ftp.metagenomics.anl.gov/data/manual/mg-rast-manual.pdf

[B94] Wright J. J. Konwar K. M. Hallam S. J. (2012). Microbial ecology of expanding oxygen minimum zones. Nat. Rev. Microbiol. 10, 381–394. 10.1038/nrmicro2778 22580367

