# Supplementary material for: Bacterioplankton Dynamics within a Large Anthropogenically Impacted Urban Estuary
Source: Front Microbiol. 2016 Jan 26;6:1438. doi: 10.3389/fmicb.2015.01438 (PMC4726783; doi:10.3389/fmicb.2015.01438)
Supplement: Supplementary file 1 [file Table1.PDF]

Supplementary Material Table 1. BEST/DistLM analysis of environmental variables.

DistLM – SEQUENTIAL TEST RESULTS:

Variable	AICc	SS(trace)	Pseudo-F	P	Prop.	Cumul.	res.df
+Temp	358.74	9625.9	14.725	0.001	0.21743	0.21743	53
+PO4	353.57	4365.4	7.4966	0.001	9.8604E-2	0.31603	52
+pH	350.91	2629.1	4.849	0.001	5.9384E-2	0.37542	51
+Sal	349.75	1745.1	3.368	0.001	3.9417E-2	0.41483	50
+ODO	349.62	1217.8	2.417	0.003	2.7507E-2	0.44234	49
+Si	349.61	1158.8	2.3639	0.001	2.6175E-2	0.46851	48

BEST SOLUTION

AICc	R^2	RSS	No.Vars	Selections
349.61	0.46851	23530	6	1-3,5,7,11
