# Supplementary material for: Bacterioplankton Dynamics within a Large Anthropogenically Impacted Urban Estuary
Source: Front Microbiol. 2016 Jan 26;6:1438. doi: 10.3389/fmicb.2015.01438 (PMC4726783; doi:10.3389/fmicb.2015.01438)
Supplement: Supplementary file 2 [file Table2.PDF]

Supplementary Material Table 2. SIMPER analysis of taxonomic groups (phylum level) driving Bray Curtis dissimilarity between February and September. Average abundance is square root transformed. Drivers of top 90% dissimilarity shown. Average dissimilarity = 19.37.

	Group september	Group february				
Species	Av.Abund	Av.Abund	Av.Diss	Diss/SD	Contrib%	Cum.%
k__Bacteria;p__Actinobacteria	0.34	0.37	3.21	1.36	16.57	16.57
k__Bacteria;p__Cyanobacteria	0.07	0.16	2.22	1.48	11.48	28.05
k__Bacteria;p__Bacteroidetes	0.49	0.42	2.04	1.13	10.53	38.59
k__Bacteria;p__Proteobacteria	0.77	0.78	1.54	1.31	7.96	46.55
k__Bacteria;p__SAR406	0.06	0.03	0.95	1.45	4.89	51.44
k__Bacteria;p__Planctomycetes	0.1	0.1	0.93	1.36	4.81	56.25
k__Bacteria;p__Firmicutes	0.05	0.04	0.88	1.11	4.54	60.79
k__Bacteria;p__SBR1093	0	0.03	0.74	1.22	3.85	64.64
k__Bacteria;p__Verrucomicrobia	0.03	0.04	0.62	1.33	3.2	67.84
k__Bacteria;p__Chloroflexi	0.02	0.02	0.59	1.12	3.06	70.9
k__Bacteria;p__Chlamydiae	0.01	0.03	0.59	1.34	3.05	73.96
k__Archaea;p__Euryarchaeota	0.02	0.01	0.51	0.87	2.63	76.59
No blast hit;Other	0.02	0.01	0.46	1.09	2.4	78.98
k__Bacteria;p__OD1	0.01	0.02	0.42	1	2.16	81.15
k__Bacteria;p__Acidobacteria	0.01	0.01	0.37	1.02	1.9	83.05
k__Bacteria;p__Gemmatimonadetes	0.01	0.01	0.36	0.94	1.85	84.89
k__Bacteria;p__Fusobacteria	0.01	0.01	0.29	0.75	1.5	86.39
k__Bacteria;p__GN02	0.01	0.01	0.25	0.72	1.31	87.7
k__Bacteria;p__Tenericutes	0	0.01	0.25	0.63	1.3	89.01
k__Bacteria;p__WS3	0.01	0	0.23	0.68	1.18	90.19
