# Supplementary material for: Bacterioplankton Dynamics within a Large Anthropogenically Impacted Urban Estuary
Source: Front Microbiol. 2016 Jan 26;6:1438. doi: 10.3389/fmicb.2015.01438 (PMC4726783; doi:10.3389/fmicb.2015.01438)
Supplement: Supplementary file 3 [file Table3.PDF]

Supplementary Material Table 3. SIMPER analysis of taxonomic groups (family level) driving Bray Curtis dissimilarity between February and September. Average abundance is square root transformed. Top 30% dissimilarity shown. Average dissimilarity = 43.35.

	Group september	Group february				
Species	Av.Abund	Av.Abund	Av.Diss	Diss/SD	Contrib%	Cum.%
k__Bacteria;p__Actinobacteria;c__Actinobacteria;o__Actinomycetales;f__Microbacteriaceae	0.14	0.27	1.61	1.29	3.71	3.71
k__Bacteria;p__Proteobacteria;c__Gammaproteobacteria;o__Oceanospirillales;f__Halomonadaceae	0.32	0.2	1.38	1.37	3.19	6.9
k__Bacteria;p__Actinobacteria;c__Acidimicrobiia;o__Acidimicrobiales;f__OCS155	0.27	0.17	1.16	1.4	2.67	9.57
k__Bacteria;p__Proteobacteria;c__Alphaproteobacteria;o__Rhodobacterales;f__Rhodobacteraceae	0.46	0.56	1.06	1.52	2.44	12.01
k__Bacteria;p__Bacteroidetes;c__Flavobacteriia;o__Flavobacteriales;f__Flavobacteriaceae	0.42	0.32	0.93	1.28	2.15	14.16
k__Bacteria;p__Bacteroidetes;c__[Saprospirae];o__[Saprospirales];f__Saprospiraceae	0.02	0.11	0.82	1.87	1.9	16.06
k__Bacteria;p__Proteobacteria;c__Gammaproteobacteria;o__Oceanospirillales;f__	0.09	0.05	0.77	0.82	1.77	17.83
k__Bacteria;p__Cyanobacteria;c__Synechococcophycideae;o__Synechococcales;f__Synechococcaceae	0.03	0.11	0.74	1.71	1.71	19.53
k__Bacteria;p__Proteobacteria;c__Alphaproteobacteria;o__;f__	0.21	0.17	0.63	1.32	1.45	20.98
k__Bacteria;p__Actinobacteria;c__Actinobacteria;o__Actinomycetales;f__	0.02	0.09	0.61	2.05	1.4	22.39
k__Bacteria;p__Proteobacteria;c__Gammaproteobacteria;o__Oceanospirillales;f__SUP05	0.06	0.11	0.59	1.35	1.35	23.74
k__Bacteria;p__Proteobacteria;c__Gammaproteobacteria;o__Thiotrichales;f__Piscirickettsiaceae	0.11	0.05	0.58	1.54	1.34	25.07
k__Bacteria;p__Proteobacteria;c__Gammaproteobacteria;o__Alteromonadales;f__OM60	0.19	0.14	0.57	1.38	1.32	26.39
k__Bacteria;p__Proteobacteria;c__Alphaproteobacteria;o__Rickettsiales;f__Pelagibacteraceae	0.1	0.14	0.56	1.76	1.3	27.69
k__Bacteria;p__Proteobacteria;c__Betaproteobacteria;o__Rhodocyclales;f__Rhodocyclaceae	0.07	0.08	0.53	1.02	1.21	28.91
k__Bacteria;p__Proteobacteria;c__Gammaproteobacteria;o__Alteromonadales;f__Alteromonadaceae	0.1	0.04	0.51	1.67	1.18	30.09
k__Bacteria;p__Bacteroidetes;c__Flavobacteriia;o__Flavobacteriales;f__Cryomorphaceae	0.19	0.15	0.49	1.06	1.13	31.22
