# Supplementary material for: Bacterioplankton Dynamics within a Large Anthropogenically Impacted Urban Estuary
Source: Front Microbiol. 2016 Jan 26;6:1438. doi: 10.3389/fmicb.2015.01438 (PMC4726783; doi:10.3389/fmicb.2015.01438)
Supplement: Supplementary file 4 [file Table4.PDF]

Supplementary Material Table 4. SIMPER analysis of taxonomic groups (family level) driving Bray Curtis dissimilarity between Marine and West/River samples. Average abundance is square root transformed. Top 35% dissimilarity shown. Average dissimilarity = 44.17.

| Species | Group WEST/RIVER Av.Abund | Group MARINE Av.Abund | Av.Diss | Diss/SD | Contrib% | Cum.% |
|---|---|---|---|---|---|---|
| k__Bacteria;p__Actinobacteria;c__Actinobacteria;o__Actinomycetales;f__Microbacteriaceae | 0.39 | 0.22 | 1.95 | 1.87 | 4.41 | 4.41 |
| k__Bacteria;p__Proteobacteria;c__Gammaproteobacteria;o__Oceanospirillales;f__Halomonadaceae | 0.06 | 0.25 | 1.69 | 2.41 | 3.82 | 8.23 |
| k__Bacteria;p__Actinobacteria;c__Acidimicrobiia;o__Acidimicrobiales;f__OCS155 | 0.09 | 0.21 | 1.14 | 1.99 | 2.59 | 10.82 |
| k__Bacteria;p__Bacteroidetes;c__Flavobacteriia;o__Flavobacteriales;f__Flavobacteriaceae | 0.23 | 0.36 | 1.13 | 2.17 | 2.55 | 13.37 |
| k__Bacteria;p__Proteobacteria;c__Alphaproteobacteria;o__;f__ | 0.1 | 0.21 | 0.95 | 1.76 | 2.14 | 15.51 |
| k__Bacteria;p__Proteobacteria;c__Alphaproteobacteria;o__Rickettsiales;f__Pelagibacteraceae | 0.07 | 0.17 | 0.87 | 2.24 | 1.97 | 17.48 |
| k__Bacteria;p__Proteobacteria;c__Gammaproteobacteria;o__Oceanospirillales;f__ | 0.11 | 0.02 | 0.87 | 0.84 | 1.97 | 19.45 |
| k__Bacteria;p__Proteobacteria;c__Alphaproteobacteria;o__Rhodobacterales;f__Rhodobacteraceae | 0.62 | 0.54 | 0.87 | 1.41 | 1.96 | 21.41 |
| k__Bacteria;p__Proteobacteria;c__Gammaproteobacteria;o__Chromatiales;f__ | 0.13 | 0.05 | 0.75 | 0.81 | 1.69 | 23.1 |
| k__Bacteria;p__Proteobacteria;c__Betaproteobacteria;o__Rhodocyclales;f__Rhodocyclaceae | 0.12 | 0.07 | 0.71 | 1.27 | 1.6 | 24.71 |
| k__Bacteria;p__Cyanobacteria;c__Synechococcophycideae;o__Synechococcales;f__Synechococcaceae | 0.07 | 0.13 | 0.7 | 1.59 | 1.58 | 26.28 |
| k__Bacteria;p__Bacteroidetes;c__[Saprospirae];o__[Saprospirales];f__Saprospiraceae | 0.11 | 0.12 | 0.57 | 1.44 | 1.3 | 27.58 |
| k__Bacteria;p__Proteobacteria;c__Gammaproteobacteria;o__Alteromonadales;f__OM60 | 0.09 | 0.15 | 0.57 | 1.8 | 1.29 | 28.87 |
| k__Bacteria;p__Cyanobacteria;c__Chloroplast;o__Stramenopiles;f__ | 0.12 | 0.08 | 0.53 | 0.99 | 1.2 | 30.07 |
| k__Bacteria;p__Proteobacteria;c__Alphaproteobacteria;o__Sphingomonadales;f__Erythrobacteraceae | 0.1 | 0.04 | 0.52 | 2.1 | 1.18 | 31.25 |
| k__Bacteria;p__Proteobacteria;c__Betaproteobacteria;o__Burkholderiales;f__Comamonadaceae | 0.07 | 0.05 | 0.51 | 1.24 | 1.15 | 32.4 |
| k__Bacteria;p__Proteobacteria;c__Gammaproteobacteria;o__Oceanospirillales;f__Oceanospirillaceae | 0.1 | 0.08 | 0.49 | 1.27 | 1.12 | 33.52 |
| k__Bacteria;p__Bacteroidetes;c__Sphingobacteriia;o__Sphingobacteriales;f__ | 0.11 | 0.06 | 0.46 | 1.6 | 1.04 | 34.56 |
| k__Bacteria;p__Bacteroidetes;c__[Rhodothermi];o__[Rhodothermales];f__[Balneolaceae] | 0.01 | 0.07 | 0.45 | 1.45 | 1.02 | 35.58 |
