# Supplementary material for: Bacterioplankton Dynamics within a Large Anthropogenically Impacted Urban Estuary
Source: Front Microbiol. 2016 Jan 26;6:1438. doi: 10.3389/fmicb.2015.01438 (PMC4726783; doi:10.3389/fmicb.2015.01438)
Supplement: Supplementary file 5 [file Image1.PDF]

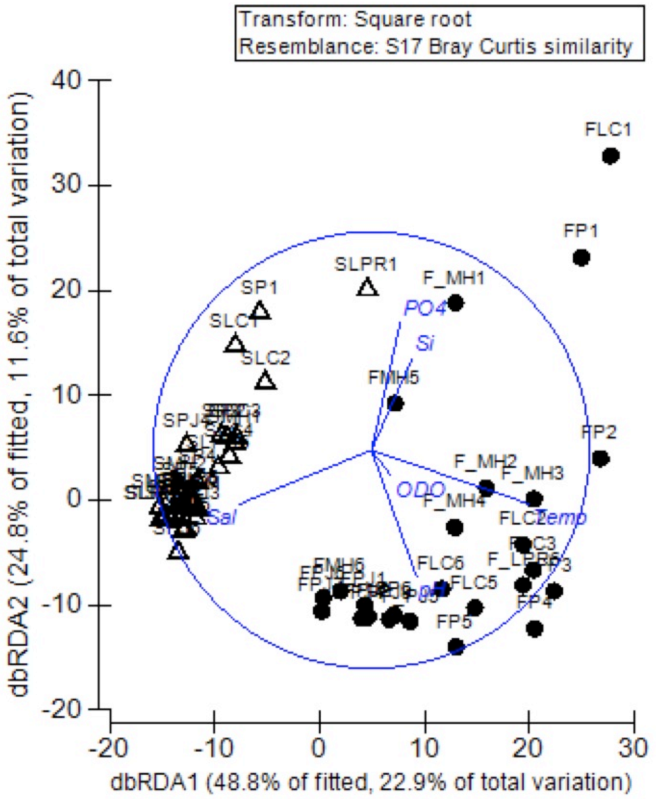

Supplementary Material Figure 1. Redundancy analysis of environmental variables and taxonomic composition. Community data is square root transformed.
