# Supplementary material for: Bacterioplankton Dynamics within a Large Anthropogenically Impacted Urban Estuary
Source: Front Microbiol. 2016 Jan 26;6:1438. doi: 10.3389/fmicb.2015.01438 (PMC4726783; doi:10.3389/fmicb.2015.01438)
Supplement: Supplementary file 9 [file Image5.PDF]

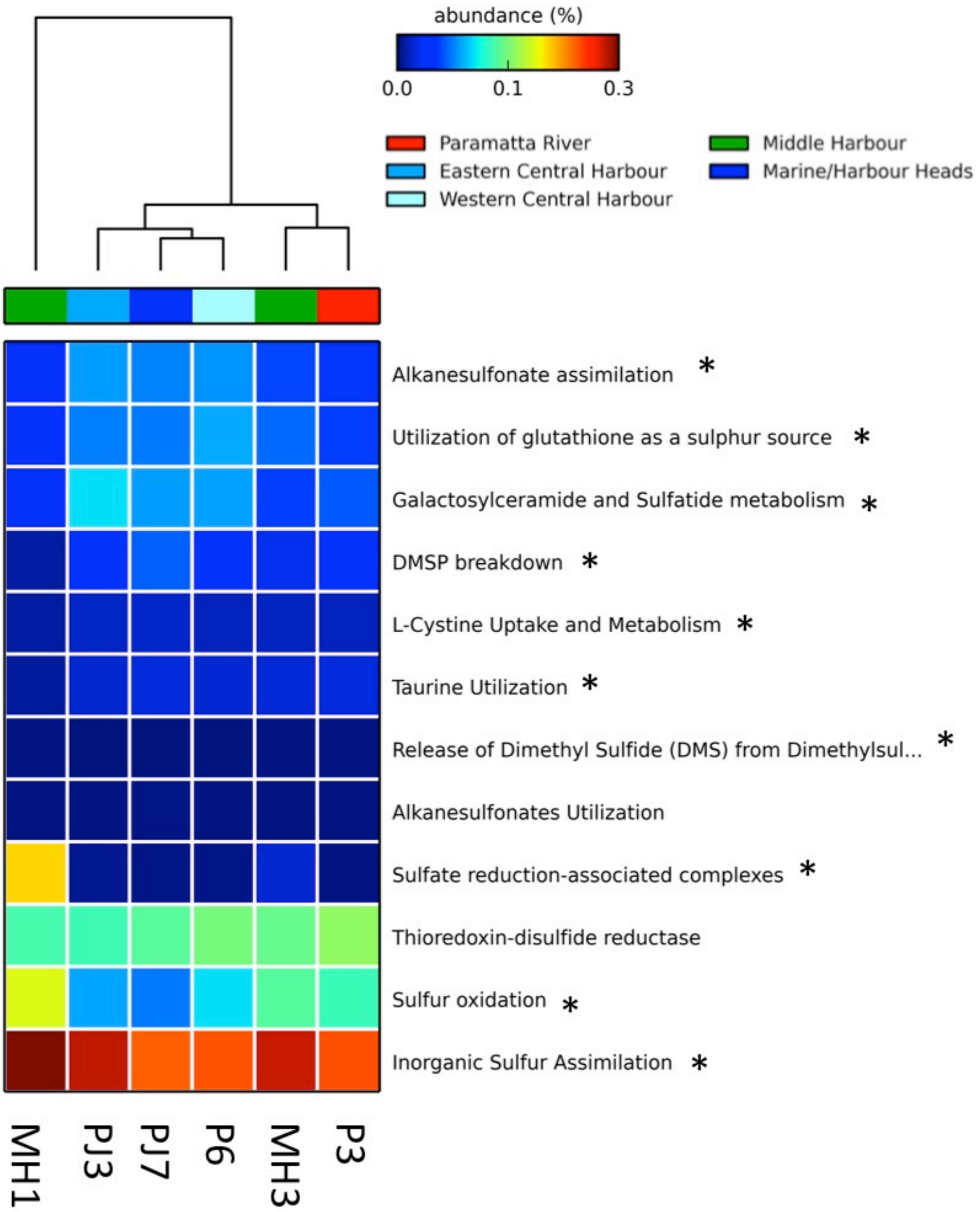

Supplementary Material Figure 5: Relative abundance of functional pathways involved in sulphur cycling at level three of the SEED hierarchy. Dendrogram clustering represents the Bray-Curtis similarity of profiles. * indicates $q<0.05$ from pair-wise comparison between PJ7 and MH1 (Fisher's exact test, Benjamini FDR multiple test correction).
