# Supplementary material for: Bacterioplankton Dynamics within a Large Anthropogenically Impacted Urban Estuary
Source: Front Microbiol. 2016 Jan 26;6:1438. doi: 10.3389/fmicb.2015.01438 (PMC4726783; doi:10.3389/fmicb.2015.01438)
Supplement: Supplementary file 10 [file Image6.PDF]

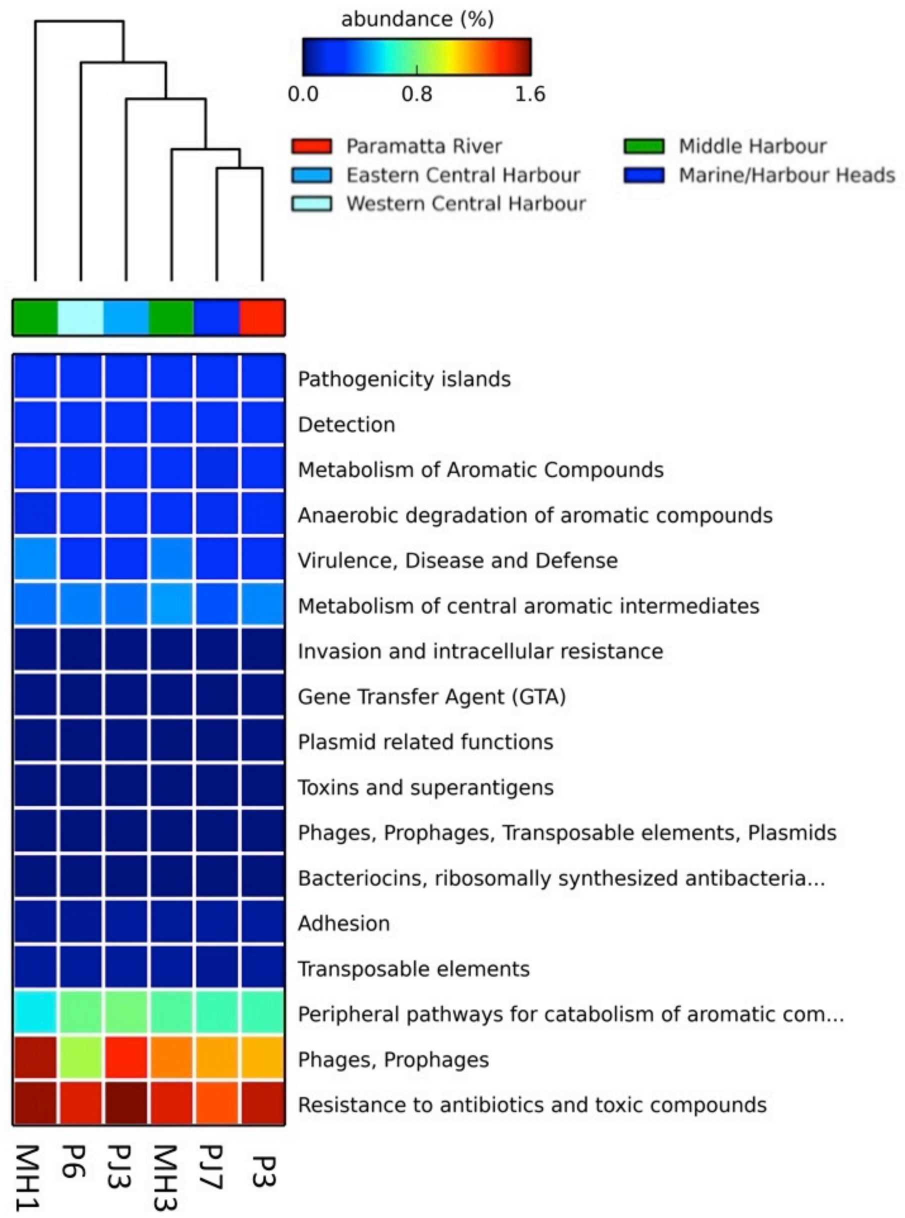

Supplementary Material Figure 6: Heatmap displaying relative abundance of functional pathways involved in virulence and degradation of aromatic compounds at level two of the SEED hierarchy. Dendrogram clustering represents the Bray-Curtis similarity of profiles.
