# Supplementary material for: Bacterioplankton Dynamics within a Large Anthropogenically Impacted Urban Estuary
Source: Front Microbiol. 2016 Jan 26;6:1438. doi: 10.3389/fmicb.2015.01438 (PMC4726783; doi:10.3389/fmicb.2015.01438)
Supplement: Supplementary file 11 [file Image7.PDF]

a)

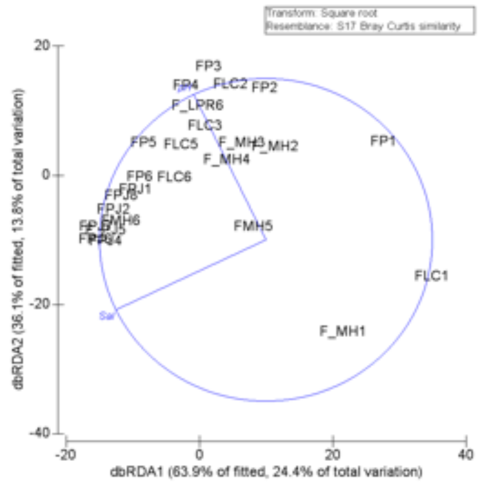

DistLM Sequential tests for February sampling period

Variable	AICc	SS(trace)	Pseudo-F	P	Prop.	Cumul.	res.df
+Sal	162.42	4227.9	7.0354	0.001	0.23424	0.23424	23
+pH	159.66	2666.2	5.2581	0.001	0.14772	0.38195	22
**BEST**							
AICc	R^2	RSS					
159.66	0.38195	11156					

b)

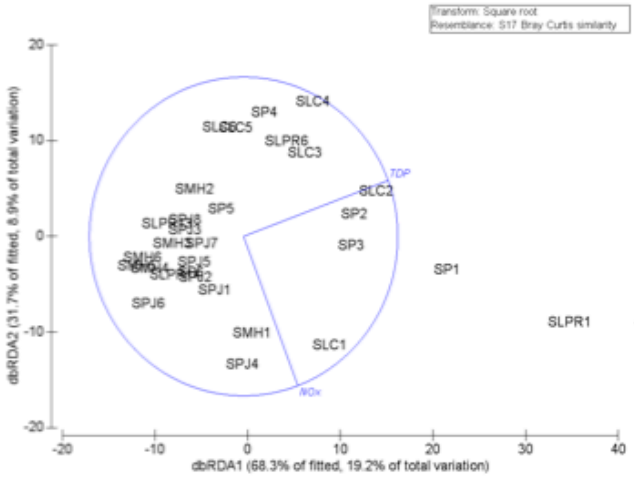

DistLM Sequential tests for September sampling period

Variable	AICc	SS(trace)	Pseudo-F	P	Prop.	Cumul.	res.df
+TDP	188.5	3293.7	6.6554	0.001	0.19204	0.19204	28
+NOx	187.46	1533.6	3.36	0.001	8.94E-02	0.28146	27
**BEST**							
AICc	R^2	RSS					
187.46	0.28146	12323					

Supplementary Material Figure 7. Redundancy analysis of environmental variables and taxonomic composition for February (a) and September (b). Community data is square root transformed. DistLM tables present only significant environmental variables for each month.
