# Supplementary material for: Bacterioplankton Dynamics within a Large Anthropogenically Impacted Urban Estuary
Source: Front Microbiol. 2016 Jan 26;6:1438. doi: 10.3389/fmicb.2015.01438 (PMC4726783; doi:10.3389/fmicb.2015.01438)
Supplement: Supplementary file 13 [file Image9.pdf]

a)

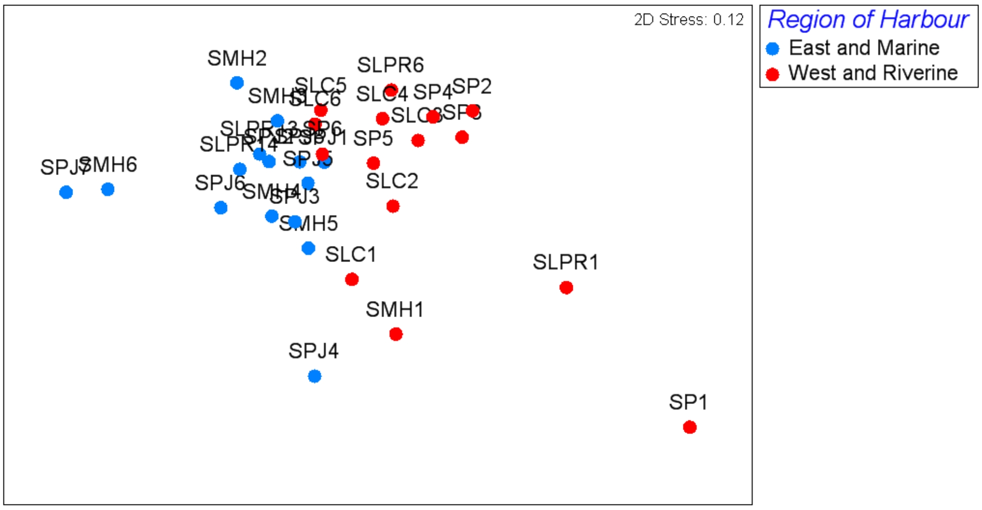

b)

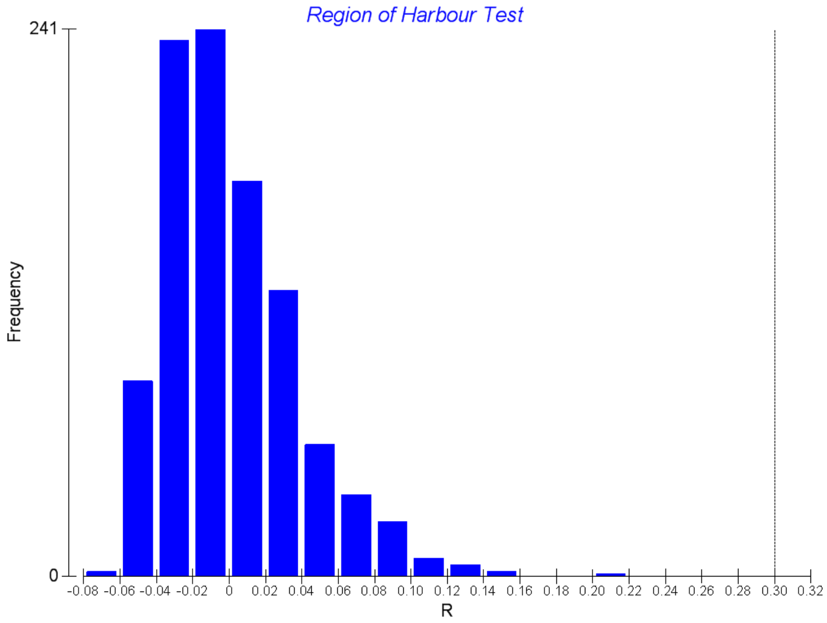

Supplementary Material Figure 9: a) Multidimensional Scaling plot of samples clustering by harbour region for September. The “East and Marine” group incorporates the regions “Eastern-central Harbour”,” Middle Harbour” and “Marine/Harbour Heads” from figures within the manuscript. West and riverine incorporates the “River”, "Western Central” and “Parramatta” groupings. b) ANOSIM analysis of grouping between Eastern and Western regions of the harbour.
