# Supplementary material for: Bacterioplankton Dynamics within a Large Anthropogenically Impacted Urban Estuary
Source: Front Microbiol. 2016 Jan 26;6:1438. doi: 10.3389/fmicb.2015.01438 (PMC4726783; doi:10.3389/fmicb.2015.01438)
Supplement: Supplementary file 14 [file Image10.pdf]

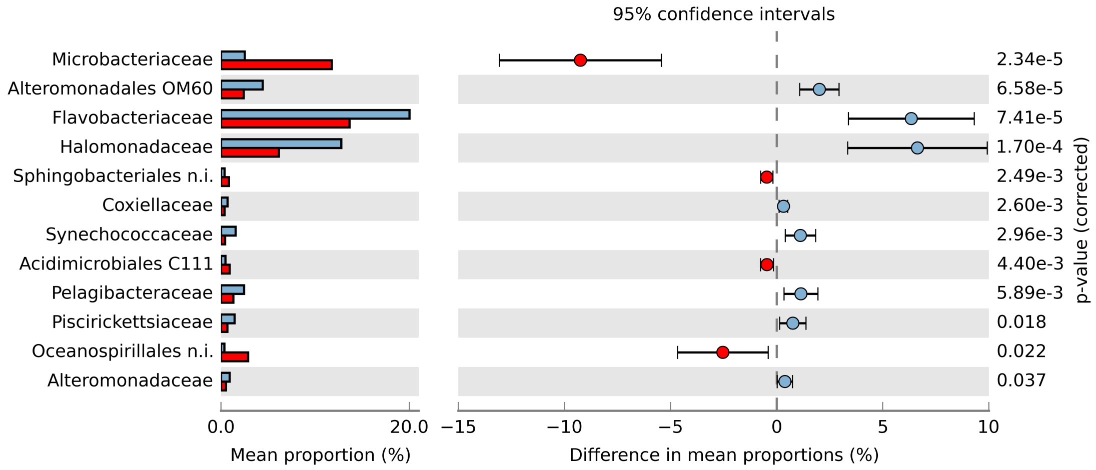

Supplementary Information Figure 10. Pairwise comparison of taxonomic profiles (family level) between Marine and Western groupings. The eastern group (blue circles) incorporates the regions "Eastern-central Harbour"," Middle Harbour" and "Marine/Harbour Heads" from figures within the manuscript. West (red circles) incorporates the "River", "Western Central" and "Parramatta" groupings. P-value is determined using Welch's T-test.
